# Aerial manipulation of long objects using adaptive neuro-fuzzy controller under battery variability

**DOI:** 10.1038/s41598-025-94937-8

**Published:** 2025-03-29

**Authors:** Praveen Kumar Muthusamy, Mohammed Basheer Mohiuddin, Anees Peringal, Bhivraj Suthar, Rajkumar Muthusamy, Irfan Hussain, Lakmal Seneviratne, Yahya Zweiri

**Affiliations:** 1 Khalifa University Center for Autonomous Robotic Systems (KUCARS), Khalifa University, Abu Dhabi, UAE; 2 Department of Mechanical & Nuclear Engineering, Khalifa University, Abu Dhabi, UAE; 3 School of Artificial Intelligence and Data Science, Indian Institute of Technology (IIT) Jodhpur, Jodhpur, India; 4 Robotics Lab, Dubai Future Labs, Dubai, UAE; 5 Department of Aerospace Engineering, Khalifa University, Abu Dhabi, UAE; 6 Interdisciplinary Research Centre for Aviation and Space Exploration (IRC-ASE), King Fahd University of Petroleum and Minerals (KFUPM), Dhahran, Saudi Arabia

**Keywords:** Quadrotor, Flight control, Fuzzy neural network, Two finger gripper, Wind disturbance, Brain emotional learning, Aerospace engineering, Electrical and electronic engineering

## Abstract

Aerial manipulation provides adaptable solutions for executing tasks in constrained environments, particularly in civil engineering and disaster response. This paper presents a UAV-based aerial manipulation system for the precise handling and transportation of long objects, such as pipes, in uncertain conditions. Compared to single- or dual-arm systems, which are difficult to scale and maintain, the proposed design features a modular two-finger gripper, enhancing scalability and reliability while reducing mechanical complexity. To address challenges such as positional drift, which can compromise mission success, the system employs a SO-BFBEL controller controller to enhance stability and precision and it is compared with DNN-MRFT-based PID and Fuzzy SMC controller. Experimental results demonstrate that the SO-BFBEL controller reduces the position tracking error up to 50% and compensates for wind disturbances and battery discharge fluctuations more effectively than conventional methods. Additionally, the SO-BFBEL controller can help to conserve battery life during manipulation phases which can boost the operational efficiency without incurring any additional costs.

## Introduction

Aerial manipulation presents transformative potential in fields ranging from civil engineering to disaster response, offering new ways to handle challenging tasks in constrained and hard-to-reach areas. The integration of unmanned aerial vehicles (UAVs) in civil engineering applications has witnessed a substantial surge owing to their versatility and adaptability to various operational scenarios. A particularly challenging and critical domain within civil engineering involves constrained areas where conventional machinery faces limitations of restricted access and constrained manoeuvrability. In this scenario, the use of UAVs equipped with aerial manipulation capabilities emerges as a promising solution^1,2^. The need for innovative solutions in civil engineering arises from the increasing demand for efficient and cost-effective methodologies to execute manipulation tasks in windy conditions. To perform aerial manipulation, it is necessary to achieve stability and robust control of the aerial manipulation platform. Quadcopter UAVs (or any rotorcraft UAVs) are more suitable for this task due to their vertical-take-off and landing (VTOL) capabilities which enables them for hovering at a certain location. Quadcopters are under-actuated non-linear systems with six degrees-of-freedom (6DOF) but only four actuators which makes their flight dynamics more complex and controlling it for aerial manipulation more challenging. Various controllers have been designed to control the quadcopter and some of the recent designs for aerial manipulation includes sliding mode controller^3^, backstepping controller^4,5^ and fuzzy logic controller^6^.

The performance of aerial manipulation systems can be severely impacted by gripper dynamics which influence payload stability and flight manoeuvrability, requiring precise compensatory control strategies. Additionally, longer payload objects introduce greater aerodynamic effects and inertial coupling and external factors such as wind pose challenges in maintaining the stability and accuracy during operation. Wind disturbances significantly impact UAV based manipulation by inducing random perturbations in both the UAV’s flight stability and grasping accuracy. Strong gusts can alter the object’s orientation mid-flight, making precise placement challenging. Wind-induced oscillations in the UAV’s attitude can propagate to the end-effector, affecting grasping precision and potentially causing task failure. Additionally, fluctuating wind patterns introduce unpredictable forces on the payload, requiring dynamic adjustments in control inputs to maintain positional accuracy. Without a robust compensation strategy, these factors can lead to deviations in trajectory tracking, unintended rotations, and loss of payload control, ultimately compromising mission reliability. This necessitates a control strategy capable of real-time adaptation and disturbance rejection.

Aerial manipulation have been performed using a UAV with single^7,8^ and multiple arm^9^ which involves complex multi-joints configurations and mechanisms to grasp an object. Aerial manipulation using multiple UAVs using suspended cable connections and their combinations are simulated using a simulator^10^ and summarised in the recent literature^11^. These multi-UAV mechanisms are quite complex due to the coordination between the UAVs as well as the grasping object. These designs also poses a risk of collision due to unbalanced center-of-gravity (CoG) of the object which can affect the stability of the system and potentially increase the cost. A survey paper on the effects of gripper actuation on aerial manipulation^12^ and their benchmarks^13^ gives more details and insights to these problems.

Aerial manipulation systems with complex mechanisms, such as single- or double-link arms, face significant challenges in operational efficiency, scalability, and maintenance, complicating their deployment in real-world settings. For effective deployment in practical applications, a scalable, modular, and robust system design is essential, with ease of operation and maintenance being paramount. Additionally, to enable outdoor use, the system must be resilient to external disturbances like wind, which can significantly affect performance and reliability. While some research addresses the general challenges of aerial manipulation, limited studies focus on the specific demands of handling long objects-such as pipes-in civil engineering environments where high wind conditions are common.

This study addresses these gaps by developing a streamlined aerial manipulation system that combines modularity and robustness to improve real-world viability. The proposed aerial manipulation system develops a customised gripper design actuated by a single servo motor with linkage mechanism to grasp an object at a single point and it is controlled using a novel neuro-fuzzy controller. This design is chosen as it is lighter and simpler to design and implement. The two-point grasping is more complex and requires additional coordination between the two gripping points. The single point grasping offers more flexibility in terms of the orientation of the long object and allows for easier rotation and manipulation around the grasping point. The orientation or position of the long object can dynamically change due to the thrust of the quadcopter during the pickup manoeuvre so flexibility offered by the single point grasping gives a significant advantage over the rigid two-point grasping method.

The challenge in performing the aerial manipulation for long object (pipes) relies on the accuracy and reliability of the quadcopter in the presence of uncertainties due to the gripper mechanisms and external disturbances^14,15^. The flight dynamics of a quadcopter exhibit a stronger non-linear behaviour when they are closer to the ground due to ground effect. This phenomenon causes the quadcopter to drift in $X-Y$ axis while it gets closer to the ground while hovering. This makes the task of picking an object from the ground more challenging even if the exact position of the object is known. The drift due to the ground effect can cause the quadcopter to completely miss the target and fail to perform the pickup task reliably. In addition to that, the position drift significantly affects the accuracy of the quadcopter to grasp the desired point of the object. This can cause an imbalance in the CoG of the long object which in turn affects the stability of the entire system and alter the position of the long object while placing it.

Another challenge in aerial manipulation and aerial robotics in general is the uncertainties in the hardware, especially the batteries, which are not explored enough. The battery discharge rate may vary from one battery to another with same specifications depending on their usage. An overused battery may have uncertain discharge rate which may compromise the mission and affect the stability of the system in long run. This is important as the controller has to compensate the lower battery by commanding higher control signal over-time and it is not feasible to tune the control system for every individual battery profile.

These problems can be tackled by incorporating an intelligent controller that can adapt to uncertainties and non-linearities in realtime. Since it is not practical to know the uncertainties and wind disturbance all the time, this paper focuses on non-model based intelligent control systems. A novel non-model based intelligent controller named the bidirectional fuzzy brain emotional learning (BFBEL) controller is introduced in^16^ to control a UAV under payload uncertainties in real-time. This controller incorporates fuzzy inference, neural networks and a novel learning algorithm based on reinforcement learning. The controller adapts rapidly in real-time from scratch which demonstrates its adaptive capabilities under payload variability. In addition, this paper also address the main drawback in the brain emotional learning (BEL) algorithm by introducing a bidirectional brain emotional learning (BBEL) algorithm^17^ which drastically improved the stability and adaptation speed of the controller.

The BFBEL controller is further developed to self-organise its fuzzy parameters in real-time which eliminates the expert knowledge required to set up the fuzzy layers. The developed controller is called the self-organised bidirectional brain emotional learning (SO-BFBEL) controller^18^ and it performed accurate trajectory tracking under extreme wind disturbance where its computation cost is measured to be quite similar to that of a conventional PID controller. Recently, it was successfully applied to perform accurate trajectory tracking on a 32g Nano S2 Helicopter UAV with low powered Lisa CPU and it demonstrated a resilient control under wind as well as communication glitches^19^. This demonstrates its ability to work with very low computation requirements and its superior control ability compared to the conventional PID makes it an ideal candidate for aerial manipulation.

Based on the discussion above, the self-organised bidirectional brain emotional learning (SO-BFBEL) controller is chosen to perform the aerial manipulation for this work. Furthermore, this paper presents the experimental evaluation of the results conducted in a controlled environment simulating real-world civil engineering scenarios such as pipe pickup and placement in a windy environment with uncertain batteries. The performance of the developed aerial manipulation system is evaluated with the SO-BFBEL controller and two more non-model based controllers for comparison, namely, deep neural network—modified relay feedback test (DNN-MRFT) based PID controller^20,21^ and adaptive fuzzy sliding mode controller (FSMC)^22^. We chose to compare with the PID controller as it is commonly used in UAVs and robotics due to its simplicity and usability. The latest technique available to enhance the PID controller using the Deep Neural Network based Modified Relay Feedback Test (DNN-MRFT) is used for comparison as showed promising results with systematic approach. Similarly, an adaptive FSMC is used for comparison instead of a conventional SMC to benchmark against an intelligent controller where the SMC is integrated and adapted using interval type 1 fuzzy logic^22^.

The comparative evaluation of SO-BFBEL, DNN-MRFT based PID, and FSMC controllers presented in this work provides insights into the adaptive capabilities required for aerial manipulation. The SO-BFBEL controller’s ability to mitigate external disturbances and system uncertainties is assessed alongside other advanced control strategies. It is also assessed for task precision, adaptability to environmental variations, reliability and overall operational efficiency. By addressing the unique challenges posed by civil engineering work in constrained areas, this research contributes to the ongoing discourse on the integration of UAVs in civil engineering applications such as pipe pickup and placement. The contributions of this paper are given below.

1. This paper presents a novel aerial manipulation system designed for precise handling and delivery of long objects, such as pipes, using a single UAV equipped with a custom-built two-finger gripper. The modular, robust and scalable design of the system avoids the complexity of traditional single- or dual-arm setups, making it suitable for practical applications and easier to maintain and deploy in real-world scenarios.
2. To address the challenges of accuracy and reliability under uncertain conditions, this paper implements a self-organised bidirectional brain emotional learning (SO-BFBEL) controller. The performance of the SO-BFBEL controller is rigorously evaluated and compared against the DNN-MRFT based PID and the FSMC controller, highlighting its superior adaptability in mitigating wind disturbances and succeeding the mission with high accuracy and reliability.
3. A detailed characteristic analysis is conducted on DNN-MRFT based PID and the SO-BFBEL controllers, with particular emphasis on the impact of battery depletion rates on system performance. The adaptive feature of the SO-BFBEL controller demonstrated accurate trajectory tracking control even under uncertain battery discharge rates and with lower battery levels. In addition, its ability to adapt below the trim value while landing and to recover instantly to hover without any overshoot reduces the battery consumption during manipulation phases which can directly improve the operational efficiency.

The rest of this work is organised as follows, the aerial manipulation system section describes the design of the system and includes the details of the gripper mechanism, quadcopter platform and the overall process. The neuro-fuzzy controller section describes design and details of the SO-BFBEL controller. The simulation section describes the control structure and the setup of the quadcopter for simulations along with the simulation results. The experimentation section describes experimental setup, aerial manipulation test methodology and analysis of the experimental results in detail. Finally, the conclusion and the discussion section gives the conclusion of the paper and discussion for future work.

## Aerial manipulation system

**Fig. 1:**
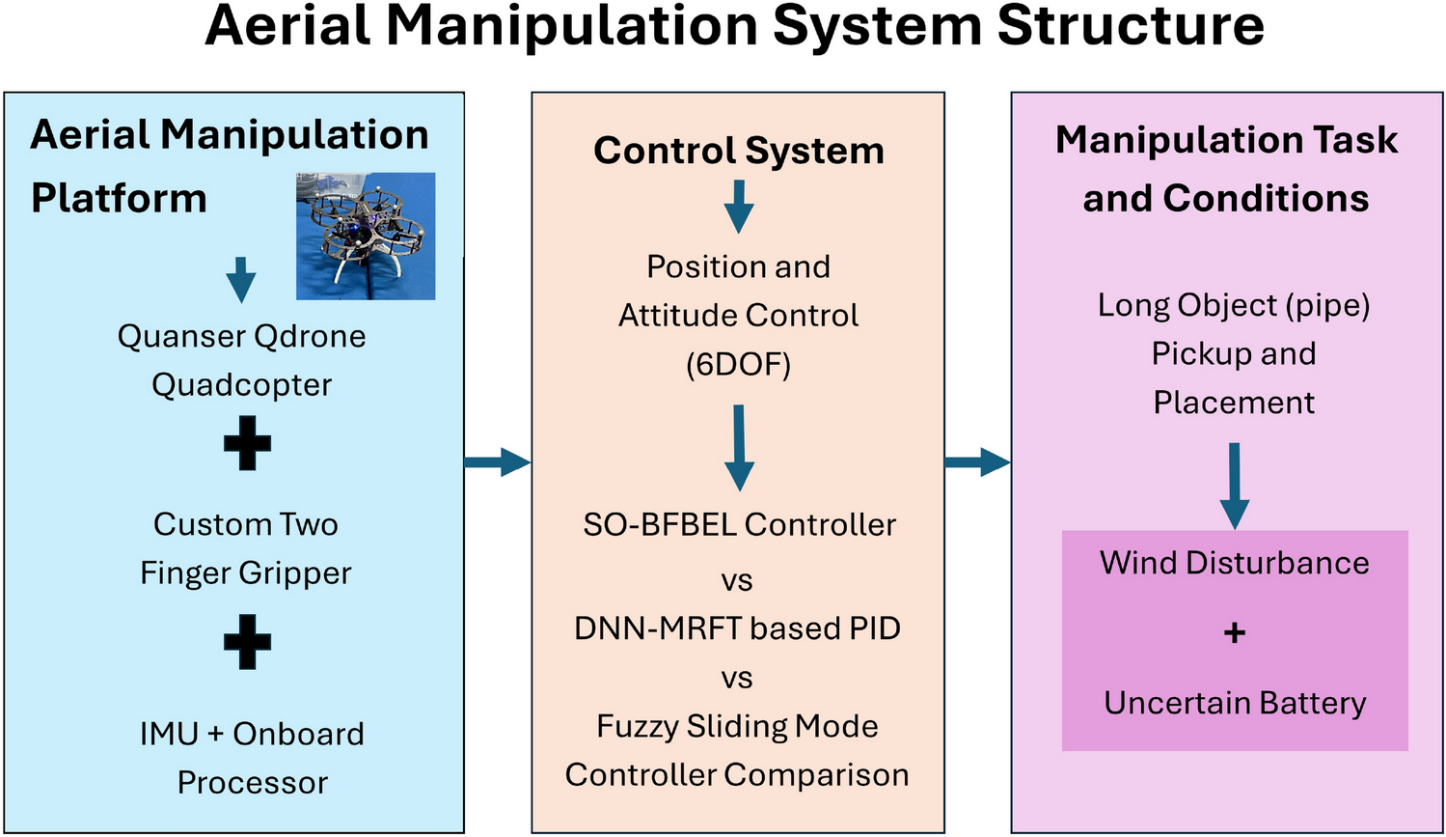
Complete overview of the proposed aerial manipulation system and its conditions.

A novel aerial manipulation system designed specifically for the efficient picking and dropping of long objects such as pipes in civil construction tasks within constrained areas subjected to extreme wind disturbance and the overview of this system is shown in Fig. 1. The system incorporates two key components with the quadcopter: a single actuator-based two-finger gripper and the SO-BFBEL control system. The integration of these elements aims to enhance the stability, adaptability, and overall performance of the system in challenging environments. The position of the quadcopter and the object is assumed to be measurable at all times.

The aerial manipulation system involves key components such as an inertial measurement unit (IMU) and an onboard computer processor. An overview of the developed aerial manipulation system is illustrated in Fig. 3. The quadcopter acts as the primary aerial platform with the payload capacity to support both the two-finger gripper and the long object (pipe). The flight controller ensures the stability of the quadcopter to perform hover and manoeuvring in three-dimensional space. The IMU provides real-time orientation and velocity data, helping to maintain the quadcopter stability while manipulating the object.

A Quanser Qdrone quadcopter is used for this work^23^ due to its capacity to add external devices for direct power and control without needing external support. The total weight of the quadcopter with the battery was measured 1151.6 g and its maximum payload is tested up to 380 g. The quadcopter has onboard Wi-Fi for communication and an Intel Aero Compute Board processor which allows to communicate and implement the control system onboard.

**Fig. 2:**
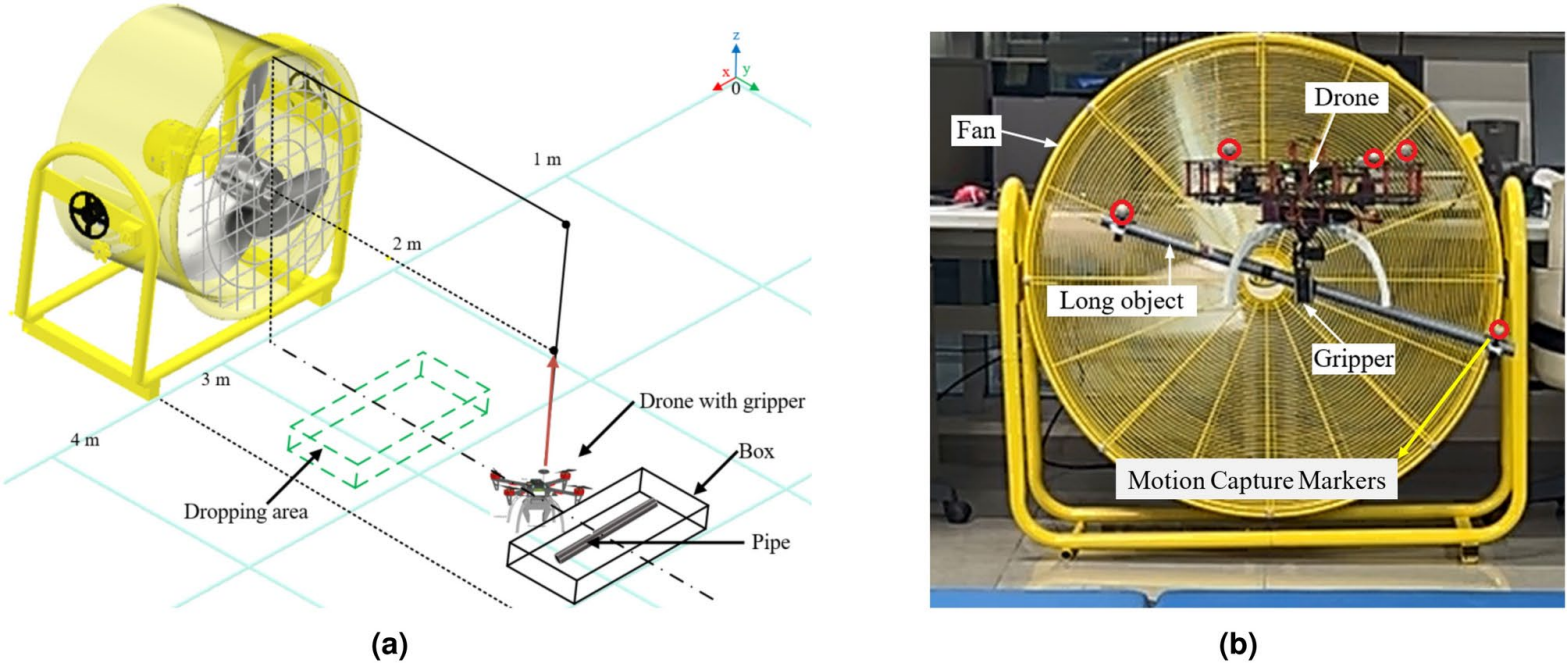
(**a**) The aerial manipulation system setup and (**b**) the aerial manipulation of long object under extreme wind disturbance.

**Fig. 3:**
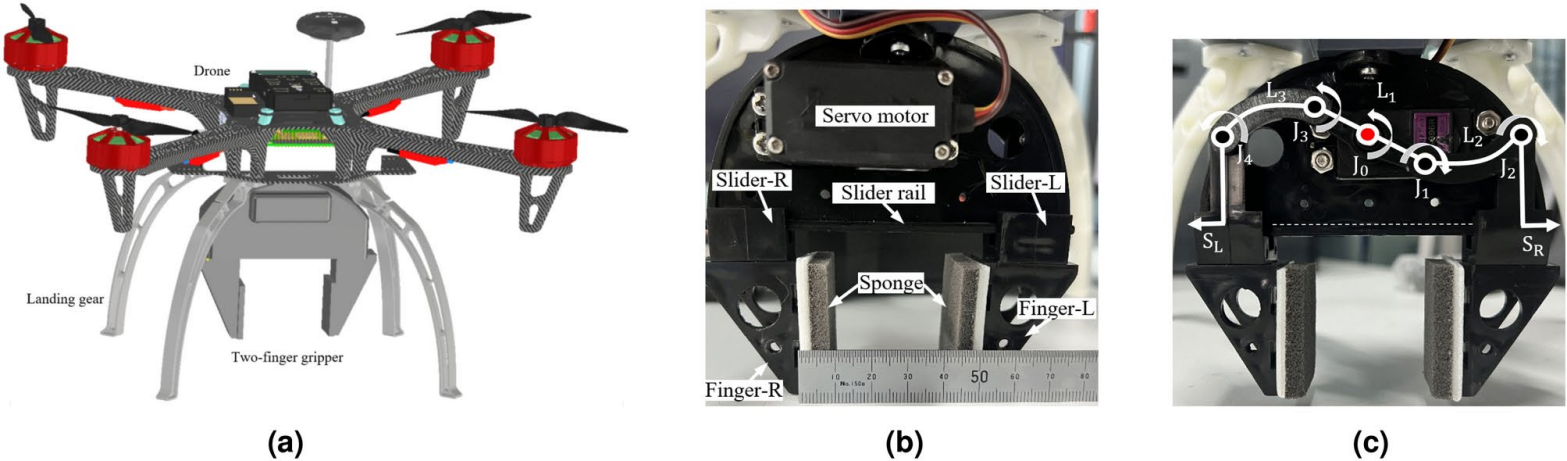
Quadcopter with two-finger gripper for aerial manipulation.

A conventional gripper design requires significant power for object grasping due to its utilisation of multiple actuators. Therefore, this paper designs a two-finger gripper operated by a single actuator, accomplished through the integration of a linkage mechanism with a servo motor and finger-sliding mechanism to grab objects effectively. The gripper is customised to fit the base of the quadcopter so that it can be modular and swapped with other platforms easily. It is then attached at CoG of the quadcopter to enable aerial manipulation tasks such as pick-and-place operations of a pipe, particularly for civil engineering applications. The gripper is made from ABS plastic material and it is manually tested to hold up to 500 g grasp load capacity. The gripper can grasp an item as small as 0.5 cm up to a maximum of 2.5 cm. The total height (length) of the gripper is 11.5 cm and the total weight of the gripper along with the landing gear attachments are 237.4 g. The overview of the aerial manipulation mission and its experimental setup is shown in Fig. 2. The entire gripper mechanism and its mechanical workings are shown in Fig. 3.

Figure 3a shows the illustration of the proposed aerial manipulation system. In Fig. 3b, it can be seen that a servo motor serves as the prime mover and this motor is intricately linked through a mechanism comprising several components. Figure 3c shows that the servo motor shaft $(J_{0})$ is connected to joint $(J_{1})$ using a link gear $(L_{1})$ with lever mechanism at both ends. The link $(L_{1})$ is directly connected to the joint $(J_{1})$ at one and end and to joint $(J_{3})$ at the other end. The joint $(J_{1})$ connects to the link $(L_{2})$ via a revolute joint at $(J_{1})$. The link $(L_{2})$, in turn, connects to the right side slider (SR) of the finger at joint $(J_{2})$. On the other end, the link gear $(L_{1})$ is linked to link $(L_{3})$ through another revolute joint at $(J_{3})$. Here, the link $(L_{3})$ connects to the left side slider (SL) of the finger via a revolute joint at $(J_{4})$. Both fingers rest upon the sliding rail to facilitate smooth opening and closing movements.

This gripper operates on an open-loop system, toggling between on and off states to grasp long objects without continuous power. Implementing closed-loop control would require a constant power supply from the quadcopter battery, reducing flight time-an undesirable outcome. Instead, the gripper uses a sliding rail mechanism, linkages, and a single actuator to mechanically lock its two fingers during grasping tasks, optimising efficiency without complex controls. A single servo motor drives a linkage attached to the fingers: rotating clockwise opens the gripper, while counter clockwise rotation closes it to grasp the object. The servo motor locks in position when unpowered, allowing the gripper to securely hold a load without drawing additional power, thereby conserving battery life and enhancing reliability. This arrangement ensures that the joint link kinematics can stabilize a load without continuous power input, crucial for UAV applications where energy efficiency is essential. By eliminating the need for a powerful battery and complex gear systems, the linkage mechanism enables the gripper to perform encompassing, spreading, and pinch grasps with a more streamlined, power-efficient design. The inner tip of both the fingers are attached with a sponge using a two sided tape to provide more grip through friction and passive envelop grasping. This attachment also dampens the vibration from the quadcopter on the object which helps to improve the grip on the object.

### Neuro-fuzzy controller

The bidirectional fuzzy brain emotional learning (BFBEL) controller is introduced in^16,24^ which has the structure of a simple fuzzy neural network (FNN) coupled with the bidirectional brain emotional learning (BBEL) algorithm introduced in^17^. This is a non-model based controller and it can be applied to any platform similar to the PID controller^25,26^. In the BFBEL controller, Gaussian membership functions are used for the fuzzy layers and they are assigned a range using the the mean and variance values and this range is called the fuzzy range. The drawback of BFBEL controller design is that range of each fuzzy layer need to be defined manually based on expert knowledge or by trial and error which is cumbersome. To address this problem a self-organising mechanism that could automatically generate or delete a new fuzzy layer with an appropriate fuzzy range in real-time was developed and introduced as the self organising bidirectional brain emotional learning (SO-BFBEL) controller in^18^ which greatly improved the capability and its ease of use. The structure of the SO-BFBEL control system is given below:

1. *Input:* The input states are defined as in Eq. (1)
  1 $$\begin{aligned} \varvec{i}=\left[ i_1, i_2,\ldots ,i_i,\ldots , i_m\right] ^T\in \Re ^m \end{aligned}$$
  where *m* is the number of input states, $\varvec{i}$ is input state vector. In this paper, $m=2$ and $\varvec{i}=[e, {\dot{e}}]$ for each individual parameter control. The SO-BFBEL controller is used to control the *X*, *Y* and *Z* position of the quadcopter.
2. *Fuzzy range:* The fuzzy range is defined using the Gaussian membership function as in Eq. (2)
  2 $$\begin{aligned} b_{ij}= \exp \left[ -\frac{1}{2} \left( \frac{i_i-\varsigma _{ij}}{\sigma _{ij}}\right) ^2\right] {\left\{ \begin{array}{ll} & \text {for} \ i=1,2,\ldots ,m\\ & \quad \ j=1,2,\ldots ,n \end{array}\right. } \end{aligned}$$
  where *n* denotes the number of fuzzy layers, $\varsigma _{ij}$ and $\sigma _{ij}$ denotes the mean and variance of the $i^{th}$ input and the $j^{th}$ layer. Here, $\sigma _{ij}$ is defined a constant value whereas $\varsigma _{ij}$ and *n* are calculated using the self-organising (SO) algorithm. The SO algorithm generates new fuzzy layers if the range covered by the existing layers does not encompass the system input. Simultaneously, it removes any existing fuzzy layers that remain unused. Activation values of the membership functions determine the decision to generate a new layer or delete an existing one. The generation of a new layer adheres to the criterion specified in Eq. (3), where $gen_l$ represents the predefined generating threshold.
  3 $$\begin{aligned} \text {if} \ \max _{i}(b_{ij})<gen_l\ \ \text {then} \ \ n_{ij}(t+1)=n_{ij}(t)+1 \end{aligned}$$
  If the condition in Eq. (3) is met, then the total number of fuzzy layers is increased, as described in Eq. (4), where $n_{ij}(t)$ denotes the number of existing fuzzy layers for the $i^{th}$ input at time *t*, and $(t+1)$ indicates the subsequent computation cycle or iteration.
  4 $$\begin{aligned} \begin{aligned} \varsigma _{ij}(t+1)&=I_i&\sigma _{ij}(t+1)&=\sigma _i&\\ v_{ij}(t+1)&=v_n&w_{ij}(t+1)&=w_n \end{aligned} \end{aligned}$$
  In Eq. (4), upon the creation of a new layer, initial values for mean ($\varsigma _{ij}(t+1)$) and variance ($\sigma _{ij}(t+1)$), along with their associated weight values ($v_{ij}(t+1)$ and $w_{ij}(t+1)$), are assigned as indicated. Here, $I_i$ represents the value of the $i^{th}$ input, $\sigma _i$ is the predefined constant, and $v_n$ and $w_n$ denote the predefined initial weight values, which are set as zero or predefined values based on the system. When the condition outlined in Eq. (4) is met, additional fuzzy layers are created, increasing the flexibility of the BFBEL controller to effectively manage the system. This expansion is essential for maintaining and improving system control. Conversely, inactive fuzzy layers are eliminated to reduce computational load. The decision to delete an existing layer is based on Eq. (5), employing a predefined deleting threshold $del_l$.
  5 $$\begin{aligned} \text {if} \ \min _{i}(b_{ij})<del_l \ \ \text {then}\ \ n_{ij}(t+1)=n_{ij}(t)-1 \end{aligned}$$
  The deletion process involves removing the layer satisfying the condition in Eq. (5), along with its associated neural network weights, thereby reducing the total number of fuzzy layers. The number of fuzzy layers is constrained within appropriate limits to prevent an excessive increase or decrease, ensuring optimal control without unnecessary complexity. These limits are set based on the requirements of the system.
3. *Dual neural networks:* Two set of neural networks are used namely the amygdala denoted by *a* and orbitofrontal cortex network denoted by *o*.
  6 $$\begin{aligned} a=\sum _{i=1}^{m}\sum _{j=1}^{n} b_{ij} v_{ij}; \qquad o =\sum _{i=1}^{m}\sum _{j=1}^{n} b_{ij} w_{ij} \end{aligned}$$
  where the notations *m* and *n* denote the number of inputs and layers, respectively. The notation $v_{ij}$ and $w_{ij}$ denotes the amygdala and the orbitofrontal cortex network weights.
4. *Output:* The output of the controller is denoted by *U* and it is defined as:
  7 $$\begin{aligned} U= a-o \end{aligned}$$
5. *Reward signal:* The reward signal (*R*) for the control system defined as:
  8 $$\begin{aligned} R =\sum _{i=1}^{m}\left( q_i i_i\right) +\left( c \ U \right) \end{aligned}$$
  where $q_i$ and *c* are the gain values of the reward signal.
6. *Weights adaptation laws:* The weights adaptation laws for both the neural networks are defined as:
  9 $$\begin{aligned} \Delta v_{ij}=\alpha \left[ b_{ij} \left( R-a)\right] \right.; \qquad \Delta w_{ij}=\beta \left[ b_{ij} \left( U-R\right) \right] \end{aligned}$$

where the adaptation laws for amygdala and orbitofrontal cortex weights are denoted by $\Delta v_{ij}$ and $\Delta w_{ij}$ and their associated learning rates are denoted by $\alpha$ and $\beta$ respectively. The weights are updated as $v_{ij}(t+1)=v_{ij}(t)+\Delta v_{ij}$ and $w_{ij}(t+1)=w_{ij}(t)+\Delta w_{ij}$.

The problem formulation and stability of the control system are the same as described in^18^ and this work complies with the same laws and boundary conditions to achieve stability.

#### Problem formulation and stability

Lets consider a first order nonlinear system described by the following equation:

10 $$\begin{aligned} \dot{\varvec{x}}(t) =\varvec{f}(\varvec{x}(t))+\varvec{g}(\varvec{x}(t))\varvec{u}(t)+\varvec{d}(t) \end{aligned}$$

where $\varvec{u}(t)\in \Re ^m$, $\varvec{x}(t)\in \Re ^m$, *m* and $\varvec{d}(t)\in \Re ^m$, denote the control signal, system output and the number of input states and the external disturbance respectively. The notations $f(\varvec{x}(t))$ and $g(\varvec{x}(t))$ represent smooth nonlinear continuous functions and they are assumed to be bounded within known limits. The nominal system is defined as

11 $$\begin{aligned} \dot{\varvec{x}}(t)=\varvec{f}_0(\varvec{x}(t))+\varvec{g}_0\varvec{u}(t) +\varvec{d}(t) \end{aligned}$$

where $\varvec{f}_0(\varvec{x}(t))$ and $\varvec{g}_0$ represent nominal $\varvec{f}(\varvec{x}(t))$ and $\varvec{g}(\varvec{x}(t))$ respectively. It is assumed that $\varvec{g}_0>0$ and it is also assumed that the nonlinear system of Eq. (11) is controllable and that $\varvec{g}_0^{-1}$ exists. With modelling uncertainties and external disturbances, the nonlinear system Eq. (10) can be written as:

12 $$\begin{aligned} \dot{\varvec{x}}(t)&= \varvec{f}_0(\varvec{x}(t))+\varvec{g}_0\varvec{u}(t)+\varvec{L_{d}}(t) \end{aligned}$$

where $\varvec{L_{d}}(t)$ is the lumped uncertainty and it denotes $\varvec{L_{d}}(t)= \varvec{l_{d}}(t) +\varvec{d}(t)$ and $\varvec{l_{d}}(t)=\Delta \varvec{f}(\varvec{x}(t))+\Delta \varvec{g}(\varvec{x}(t))$. Here, $\Delta \varvec{f}(\varvec{x}(t))$ and $\Delta \varvec{g}(\varvec{x}(t))$ denote the modelling uncertainties of $\varvec{f}(\varvec{x}(t))$ and $\varvec{g}(\varvec{x}(t))$. The control problem is to design a control system where the system output $\varvec{x}(t)$ tracks the desired trajectory $\varvec{x}_d(t)$. The tracking error is defined as

13 $$\begin{aligned} \dot{\varvec{e}}(t)= \dot{\varvec{x}}(t)-\dot{\varvec{x}_d}(t)\in \Re ^m \end{aligned}$$

Substituting Eq. (12) in Eq. (13), we get Eq. (14).

14 $$\begin{aligned} \varvec{{\dot{e}}}(t)= \varvec{f}_0(\varvec{x}(t))+\varvec{g}_0\varvec{u}+\varvec{L_{d}}(t)-\dot{\varvec{x}}_{d}(t) \end{aligned}$$

If $\varvec{L_{d}}(t)$ is zero or exactly known, an ideal controller can be designed as

15 $$\begin{aligned} \varvec{u}^*(t)=\varvec{g}_0^{-1}\Big [\dot{\varvec{x}}_{d}(t)-\varvec{f}_0(\varvec{x}(t))-\varvec{L_{d}}(t)-\varvec{h}^T\varvec{e}-\varvec{U_{r}}(t)\Big ] \end{aligned}$$

where $\varvec{h}\in \Re ^{m\times n}$ is the feedback gain matrix and its values are chosen to correspond to the coefficients of a Hurwitz polynomial, that is, a polynomial whose roots lie in the open left half of the complex plane, so that $\lim _{t \rightarrow \infty } ||\varvec{e}|| = 0$. In Eq. (15), $\varvec{L_{d}}(t)$ is assumed to be $||\varvec{L_{d}}||_1<\varvec{U_{r}}$ where $\varvec{U_{r}}$ is the approximation controller that can compensate for any approximation error and it is calculated as ${U_{r}}(t)= (2r^2)^{-1} (r^2+1) e(t)$ where *r* is the prescribed positive attenuation constant. However, uncertainty $\varvec{L_{d}}(t)$, is non-zero and generally unknown, so an ideal controller is not practical. A practical BFBEL control is proposed to achieve the desired control for nonlinear systems with uncertainties.

There exists an optimal BFBEL control $U^*_{\text {BFBEL}}$ to learn the ideal controller $U_I$ such that;

16 $$\begin{aligned} U_I&=U^*_{\text {BFBEL}}(i,\varvec{f}_i,\varvec{W}^*_a,\varvec{W}^*_o)+ \epsilon = a^*-o^*+ \epsilon \ = {f}_1\varvec{l}_a\varvec{W}^*_a- {f}_2\varvec{l}_o\varvec{W}^*_o+ \epsilon \end{aligned}$$

where ${f}_1=R-a$, ${f}_2=U_{\text {BFBEL}}-R$, $\varvec{l}_a=[b_{11},...,b_{ij}]$, $\varvec{l}_o=[b_{11},...,b_{ij}]$, $\varvec{W}_a=[v_{11},...,v_{ij}]^T$, and $\varvec{W}_o=[w_{11},...,w_{ij}]^T$. Here, $\epsilon$ is a minimum reconstructed error, $\varvec{W}^*_a$ and $\varvec{W}^*_o$ are the optimal parameters of $\varvec{W}_a$ $\varvec{W}_o$. The control law of the BFBEL scheme is assumed to take the following form

17 $$\begin{aligned} U={\hat{U}}_{\text {BFBEL}}((i,\varvec{f}_i,\varvec{{\hat{W}}}_a,\varvec{{\hat{W}}}_o))={ f}_1\varvec{l}_a\varvec{{\hat{W}}}_a- {f}_2\varvec{l}_o\varvec{{\hat{W}}}_o \end{aligned}$$

where $\varvec{{\hat{W}}}_a$ and $\varvec{{\hat{W}}}_o$ are estimates of the optimal parameters obtained from the tuning algorithm. Subtracting Eqs. (16) and (17), an approximate $\tilde{U}$ is defined as

18 $$\begin{aligned} \tilde{U}= U_I-U_{\text {BFBEL}}={f}_1\varvec{l}_a \tilde{\varvec{W}}_a- {f}_2\varvec{l}_o\tilde{\varvec{W}}_o+ \epsilon = F_1-F_2 + \epsilon ,\quad \text {where} \ F_1=f_{1}\varvec{l}_a \tilde{\varvec{W}}_a\quad \text {and} \ F_2=f_{2}\varvec{l}_o\tilde{\varvec{W}}_o \end{aligned}$$

##### Theorem 1

For a first-order nonlinear system given in Eq. (12), if the BFBEL controller is designed as in Eq. (17) and the adaptation laws of the BFBEL controller are designed as in Eqs. (19a–20b), then the convergence of the network parameters and the tracking error of the proposed BFBEL control system can be assured to be uniformly ultimately bounded (UUB).

19a $$\begin{aligned} \hat{\dot{\varvec{W}}}_a&= -\varvec{\alpha }(\varvec{e}^Tf_{1}\varvec{l}_{a})^T \qquad \text {if } (||\hat{\varvec{W}}_a||<\varvec{b_{a}}) \text { or } (||\hat{\varvec{W}}_a||=\varvec{b_{a}} \text { and } \varvec{e}^Tf_{1}\varvec{l}_{a} \hat{\varvec{W}}_a \ge 0 ) \end{aligned}$$

19b $$\begin{aligned} \hat{\dot{\varvec{W}}}_a&= -\varvec{\alpha }(\varvec{e}^Tf_{1}\varvec{l}_{a})^T+ \varvec{\alpha }[\varvec{e}^Tf_{1}\varvec{l}_{a}(\hat{\varvec{W}}_a^T\hat{\varvec{W}}_a/||\hat{\varvec{W}}_a||^2)]^T\quad \text {if } (||\hat{\varvec{W}}_a||=\varvec{b_{a}}) \text { and } (\varvec{e}^Tf_{1}\varvec{l}_{a} \hat{\varvec{W}}_a < 0) \end{aligned}$$

20a $$\begin{aligned} \hat{\dot{\varvec{W}}}_o&= \varvec{\beta }(\varvec{e}^Tf_{2}\varvec{l}_{o})^T\qquad \text {if } (||\hat{\varvec{W}}_o||<\varvec{b_{o}}) \text { or } (||\hat{\varvec{W}}_o||=\varvec{b_{o}} \text { and } f_{2}\varvec{l}_o \hat{\varvec{W}}_o \ge 0 ) \end{aligned}$$

20b $$\begin{aligned} \hat{\dot{\varvec{W}}}_o&= \varvec{\beta }(\varvec{e}^Tf_{2}\varvec{l}_{o})^T-\varvec{\beta }[\varvec{e}^Tf_{2} \varvec{l}_{o}(\hat{\varvec{W}}_o^T\hat{\varvec{W}}_o/||\varvec{{\hat{W}}}_o||^2)]^T\quad \text {if } (||\hat{\varvec{W}}_o||=\varvec{b_{o}}) \text { and } (\varvec{e}^Tf_{2}\varvec{l}_{o} \hat{\varvec{W}}_o < 0) \end{aligned}$$

Here, ||.|| denotes the Euclidean norm; $\varvec{\alpha }$ and $\varvec{\beta }$ are the learning rates; $f_{1}$ and $f_{2}$ are the amygdala and orbitofrontal cortex processing functions which incorporates positive and negative rewards; and $\varvec{b_{a}}$ and $\varvec{b_{o}}$ are the given parameter bounds.

##### Proof

Denoting $V(\varvec{i}(t),\varvec{f_{i}},\varvec{\tilde{W}_{a}},\varvec{\tilde{W}_{o}})$ as *V*, define a Lyapunov function candidate as,

$$\begin{aligned} V&= \frac{1}{2}{\varvec{e}^T \varvec{g}_0^{-1}\varvec{e}}+\frac{1}{2\varvec{\alpha }}\ tr(\tilde{\varvec{W}}^T_a\varvec{\tilde{W}_{a}})+\frac{1}{2\varvec{\beta }}\ tr(\tilde{\varvec{W}}^T_o\varvec{\tilde{W}_{o}}) \\ {\dot{V}}&=\varvec{e}^T\varvec{g}_0^{-1}{\dot{\varvec{e}}}-\frac{1}{\varvec{\alpha }}\ tr(\hat{\dot{\varvec{W}}}^T_a\varvec{\tilde{W}_{a}})-\frac{1}{\varvec{\beta }}\ tr(\hat{\dot{\varvec{W}}}^T_o\varvec{\tilde{W}_{o}}) =\varvec{e}^T\varvec{g}_0^{-1}{\dot{\varvec{e}}}-A-O \end{aligned}$$

where $$A=\frac{1}{\varvec{\alpha }}\ tr(\hat{\dot{\varvec{W}}}^T_a\varvec{\tilde{W}_{a}})$$ and $$O=\frac{1}{\varvec{\beta }}\ tr(\hat{\dot{\varvec{W}}}^T_o\varvec{\tilde{W}_{o}})$$.

By subtracting the system dynamic model in Eq. (12) from the control law in Eq. (15) we get $\varvec{{\dot{e}}}=-\varvec{g}_0\tilde{\varvec{U}}-\varvec{g}_0\varvec{he}- \varvec{g}_0 \varvec{U_{r}}(t)$ and by using Eq. (18), we can obtain:

$$\begin{aligned} {\dot{V}}&=\varvec{e}^T \varvec{g}_0^{-1}(-\varvec{g}_0\tilde{\varvec{U}}-\varvec{g}_0 \varvec{h}^T\varvec{e}-\varvec{g}_0\varvec{U_{r}}(t))-A-O =-\varvec{e}^T\tilde{\varvec{U}}-\varvec{e}^T\varvec{he}-\varvec{e}^T \varvec{U_{r}}(t)-A-O\\&=-\varvec{e}^T(F_1- F_2+ \epsilon )-\varvec{e}^T\varvec{he} -\varvec{e}^T \varvec{U_{r}}(t)-A-O =-\varvec{e}^T F_1 + \varvec{e}^T F_2- \varvec{e}^T \epsilon -\varvec{e}^T\varvec{he} -\varvec{e}^T \varvec{U_{r}}(t)-A-O\\&=-\underbrace{\left( A+ \varvec{e}^T F_1 \right) }_{\varvec{V}_a} - \underbrace{\left( O-\varvec{e}^T F_2 \right) }_{\varvec{V}_o} - \varvec{e}^T \epsilon -\varvec{e}^T\varvec{he} -\varvec{e}^T \varvec{U_{r}}(t) =-\varvec{V}_a-\varvec{V}_o- \varvec{e}^T \epsilon -\varvec{e}^T\varvec{he} -\varvec{e}^T \varvec{U_{r}}(t) \end{aligned}$$

Here the uncertain term is assumed to be bounded as $||\epsilon ||_1<{\varvec{U_{r}}}$ and ${\dot{V}}(\varvec{i}(t),\varvec{f_{i}},\varvec{\tilde{W}_{a}},\varvec{\tilde{W}_{o}})\le 0$, which is a negative semi-definite function and

$$\begin{aligned} {V}(\varvec{i}(t),\varvec{f_{i}},\varvec{\tilde{W}_{a}},\varvec{\tilde{W}_{o}})<{V}(\varvec{i}(0),\varvec{f_{i}},\hat{\varvec{W}}_a,\hat{\varvec{W}}_o) \end{aligned}$$

This implies $\varvec{i},\varvec{f},\varvec{\tilde{W}_{a}}$ and $\varvec{\tilde{W}_{o}}$ are bounded. Let function $B_b(t)\equiv -\varvec{e}^T(t) ~\varvec{h ~{\dot{e}}}(t)\le {\dot{V}}(t)$, so that

$$\begin{aligned} \int _{x_0}^{x_t} B_b(x)dx \le \int _{0}^t V(\varvec{i}(0))-V(\varvec{i}(t)) \end{aligned}$$

In Eqs. (19a) and (19b), if the corresponding situations $||\hat{\varvec{W}}_a||=\varvec{b_{a}}$ and $\varvec{e}^T f_{1}\varvec{l}_{a}\hat{\varvec{W}}_a<0$ are met then the following condition $(\varvec{W_{a}}^*-\hat{\varvec{W}}_a)\hat{\varvec{W}}_a^T=0.5(||\varvec{W_{a}}^*||^2-||\hat{\varvec{W}}_a||^2-||\hat{\varvec{W}}_a-\varvec{W_{a}}^*||)<0$ holds because $||\varvec{W_{a}}^*||<\varvec{b_{a}}$. This concludes that $\varvec{V_{a}}\ge 0$. Similarly, in Eqs. (20a) and (20b), if the corresponding situations $||\hat{\varvec{W}}_o||=\varvec{b_{o}}$ and $\varvec{e}^T f_{2}\varvec{l}_{o}\hat{\varvec{W}}_o<0$ are met then the following condition $(\varvec{W_{o}}^*-\hat{\varvec{W}}_o)\hat{\varvec{W}}_o^T=0.5(||\varvec{W_{o}}^*||^2-||\hat{\varvec{W}}_o||^2-||\hat{\varvec{W}}_o-\varvec{W_{o}}^*||)<0$ holds because $||\varvec{W_{o}}^*||<\varvec{b_{o}}$. This concludes that $\varvec{V_{o}}\ge 0$.

Because ${V}(\varvec{i}(0),\varvec{f_{i}},\varvec{\tilde{W}_{a}},\varvec{\tilde{W}_{o}})$ is bounded functions, ${V}(\varvec{i}(t),\varvec{f_{i}},\varvec{\tilde{W}_{a}},\varvec{\tilde{W}_{o}})$ is a non increasing and bounded function and $\lim _{t \rightarrow \infty }\int _{0}^t B_b(x)dx<\infty$. According to Lyapunov’s stability theorem and Barbalat’s lemma, this implies uniform ultimate boundedness (UUB) of the tracking error signal. Barbalat’s Lemma can be used here to show that the error signal is bounded. Moreover, the parameter estimation errors $\varvec{\tilde{W}_{a}},\varvec{\tilde{W}_{o}}$ can be guaranteed to be bounded in the sense of projection algorithm^27–29^. This guarantees the stability of the system.

## Fuzzy sliding mode controller (FSMC)

The conventional sliding mode controller (SMC) is derived based on the system model but for this paper a non-model based fuzzy SMC that does not need any training is designed for comparison. The details of the FSMC is given below:

The sliding surface for the control system is defined as:

21 $$\begin{aligned} s = G_e e + G_{de} {\dot{e}} \end{aligned}$$

where *e* is the error signal, ${\dot{e}}$ is the derivative of the error and $G_e$ and $G_{de}$ are control gains. The update rule for the consequent parameters is given by:

22 $$\begin{aligned} {\textbf{c}}_{\text {new}} = {\textbf{c}}_{\text {new}} + \gamma \begin{bmatrix} s \\ s e \\ s {\dot{e}} \end{bmatrix}, \quad \gamma = {\left\{ \begin{array}{ll} 0.1, & \text {if } t < 2.3 \\ 1, & \text {otherwise} \end{array}\right. } \end{aligned}$$

The firing strength for each fuzzy rule *i* is computed as:

23 $$\begin{aligned} \mu _i = \prod _{j} \text {mg}(x_j, X_{ij}), \quad y = \frac{\sum _{i} \mu _i z_i}{\sum _{i} \mu _i}, \quad z = {\textbf{c}}_{\text {new}} \begin{bmatrix} 1 \\ e \\ {\dot{e}} \end{bmatrix} \end{aligned}$$

Here, $\mu _i$ represents the firing strength of rule *i* based on the membership function evaluation $\text {mg}(x, X)$. The control output *y* is computed as a weighted sum of rule outputs $z_i$, where *z* is determined by the updated fuzzy consequent parameters ${\textbf{c}}_{\text {new}}$. The parameter $\gamma$ is an adaptation gain that adjusts the rate of updating the consequent parameters. It varies with time, ensuring a smooth transition in control adaptation. For $t < 2.3$, a lower value of $\gamma$ is used to maintain stability, while for $t \ge 2.3$, a higher value is used to improve responsiveness. The FSMC is applied to control the *XYZ* position and the DNN-MRFT based PID is used for the attitude control for this controller.

**Fig. 4:**
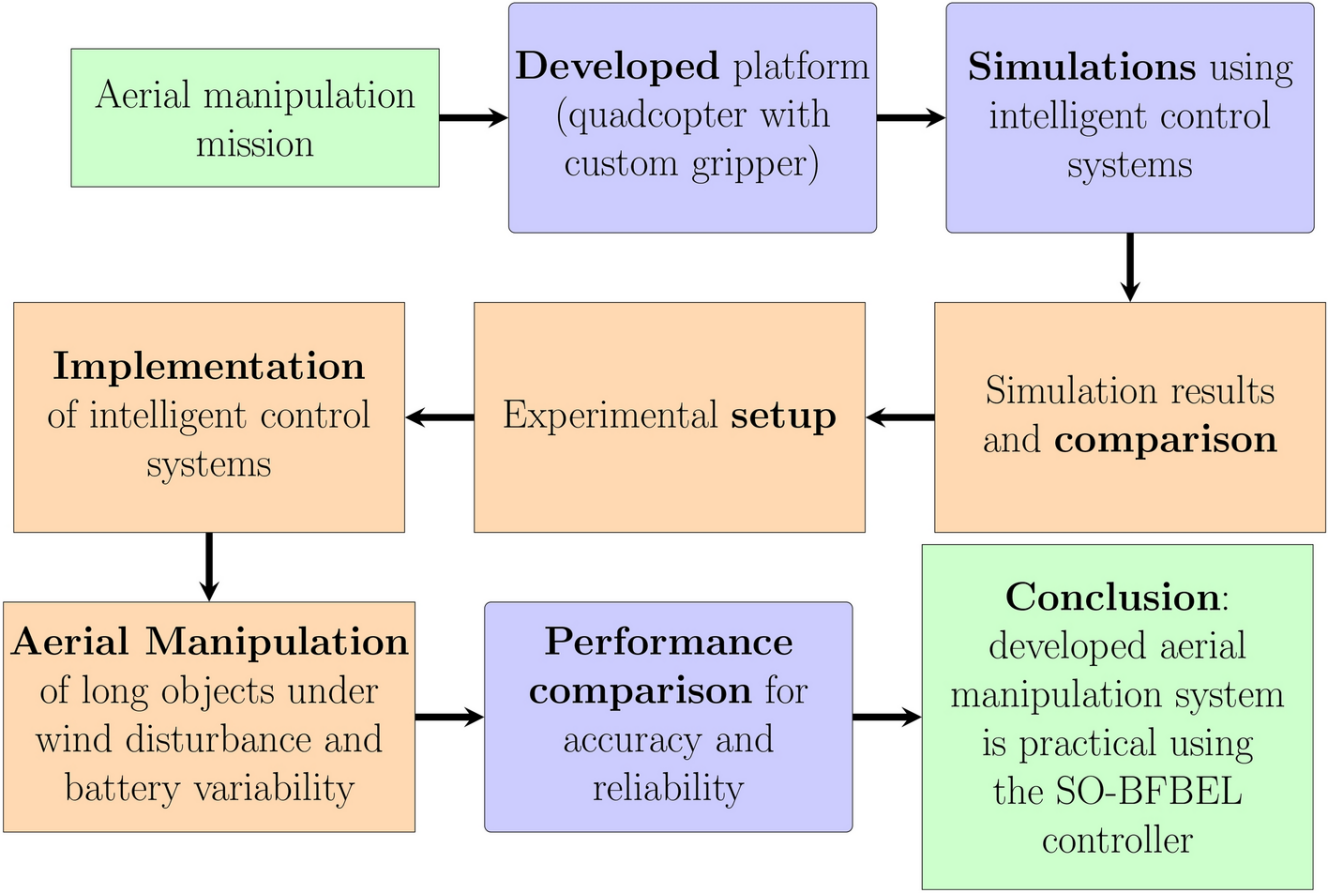
Flow chart of the proposed aerial manipulation system to complete the mission.

The overall aerial manipulation mission and the process undergone to achieve it is illustrated in Fig. 4.

## Simulations

The action of aerial manipulation using a quadcopter UAV is mimicked by simulating the quadcopter with a slung load weight where the dimension of the slung load is set the same as the gripper and the pipe load. Quadrotor slung load system entails a quadrotor UAV carrying a load beneath it through a rigid cable. The interaction between the UAV and the suspended load poses distinctive control challenges. The swinging movement of the load can destabilise the UAV, necessitating careful consideration in the control and design of the system.

To analyse the motion of the load, a straightforward pendulum model is employed, factoring in cable tension and forces on the load. The quadrotors dynamic equations must encompass the forces and moments induced by the slung load, primarily arising from cable tension and potential load swings. The dynamics of the system are formulated by addressing both the translational dynamics of the quadrotor and the angular dynamics of the load. This is based on the assumption that the suspended load is connected at CoG of the UAV. It is also assumed that the cable is rigid and the length of the slung load is set to 11.5 cm to mimic the length of the gripper hardware. The overall system is described by a set of interconnected non-linear differential equations as given in^5,24^. The equations of motion for the quadrotor with slung load are given below:

24 $$\begin{aligned} F_x&= ( \cos \phi \sin \theta \cos \psi + \sin \phi \sin \psi ) U_1 \end{aligned}$$

25 $$\begin{aligned} F_y&= ( \cos \phi \sin \theta \sin \psi + \sin \phi \cos \psi ) U_1 \end{aligned}$$

26 $$\begin{aligned} F_z&= (\cos \phi \cos \theta ) U_1 - m_l \ g \end{aligned}$$

27 $$\begin{aligned} \ddot{\phi }&=\frac{{\dot{\theta }}{\dot{\psi }}}{I_x}((I_y- I_z) + l~ U_2) \end{aligned}$$

28 $$\begin{aligned} \ddot{\theta }&=\frac{{\dot{\phi }}{\dot{\psi }}}{I_y}((I_z- I_x) + l~ U_3) \end{aligned}$$

29 $$\begin{aligned} \ddot{\psi }&=\frac{{\dot{\phi }}{\dot{\theta }}}{I_z}((I_x- I_y) + U_4) \end{aligned}$$

30 $$\begin{aligned} \ddot{x}&= \frac{F_x - m_l L \ddot{\alpha } \cos \alpha \cos \beta + m_l L {\dot{\alpha }}^2 \sin \alpha \cos \beta }{m_q + m_l} \end{aligned}$$

31 $$\begin{aligned} \ddot{y}&= \frac{F_y - m_l L \ddot{\beta } \cos \alpha \cos \beta + m_l L {\dot{\beta }}^2 \sin \beta \cos \alpha }{m_q + m_l} \end{aligned}$$

32 $$\begin{aligned} \ddot{z}&= \frac{F_z - (m_q + m_l) g}{m_q + m_l} + \frac{m_l L \ddot{\alpha } \sin \alpha \cos \beta + m_l L \ddot{\beta } \cos \alpha \sin \beta }{m_q + m_l} + \frac{m_l L {\dot{\alpha }}^2 \cos \alpha \cos \beta + m_l L {\dot{\beta }}^2 \cos \alpha \cos \beta }{m_q + m_l} \end{aligned}$$

The angular dynamics of the load with respect to the *x* and *y* axes are:

33 $$\begin{aligned} \ddot{\alpha }&= -\frac{F_x \cos \alpha + F_y \sin \alpha \sin \beta }{m_q L} -\frac{F_z \sin \alpha \cos \beta + m_q L {\dot{\beta }}^2 \sin \alpha \cos \alpha }{m_q L} \end{aligned}$$

34 $$\begin{aligned} \ddot{\beta }&= \frac{2 m_q L {\dot{\alpha }} {\dot{\beta }} \sin \alpha - F_y \cos \beta - F_z \sin \beta }{m_q L \cos \alpha } \end{aligned}$$

where, $m_q$ is mass of the quadrotor, $m_l$ is mass of the slung load, $F_{\{x,y,x\}}$ are the thrust forces along the *x*, *y*,  and *z* axes, respectively, $\alpha$ is angle of the load with respect to the x-axis, $\beta$ is angle of the load with respect to the y-axis, $L$ is length of the cable. The notations, *l* is the distance from the COG of the QUAV frame to the centre of the rotor and *d* is the drag factor. $U_1, U_2, U_3$ and $U_4$ denotes the controller output for altitude (Z-position), roll $(\phi )$, pitch $(\theta )$ and yaw $(\psi )$ respectively. The notation $I_{\{x,y,z\}}$ are the mass moment of inertia along the *x*,  *y*,  *z* axes respectively.

**Fig. 5:**
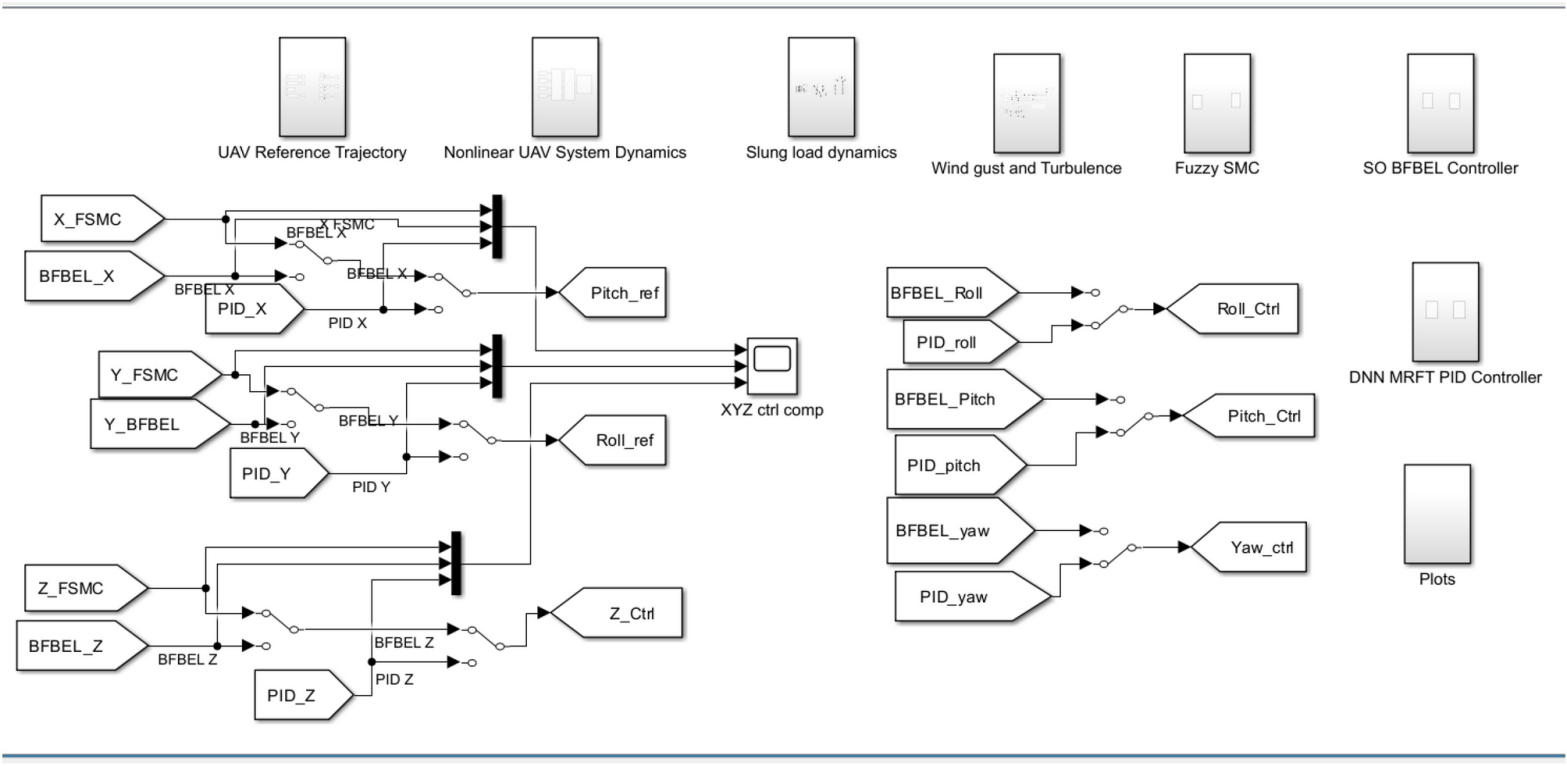
Overview of the simulation in Simulink with subsystems that denotes its specific functions such as: UAV reference trajectory, non-linear UAV system dynamics, slung load dynamics, wind gust and turbulence, fuzzy SMC, SO-BFBEL controller, DNN-MRFT PID controller and plots.

A discrete wind gust block from the Aerospace block set in Simulink is utilised to create wind gusts^30^, while a Dryden spectra model, implemented with a *S*-function block, generates realistic atmospheric turbulence and they are combined together for simulating wind disturbances^31^. The wind gust is produced with specific parameters: wind velocity = ${5}\,\hbox {m\,s}^{-1}$, gust length [*dx* *dy* *dz*] (m): [50 50 30], gust amplitude [*ug* *vg* *wg*] ($\hbox {m\,s}^{-1}$): [5 5 5]. Turbulence is generated with the following settings: turbulence scale (m) for X = Y = 22.5, Z = 3; mean wind velocity = $12\,\hbox {m\,s}^{-1}$. The wind disturbance profile has increasing variability of wind as the wind speed is raised by 2.5$\hbox {m\,s}^{-1}$ every 5 seconds, saturating around 5$\hbox {m\,s}^{-1}$ to simulate extreme scenarios. This comprehensive simulation aims to evaluate the feasibility of the control methods before transitioning to real flight tests. The overview of the entire simulation in Simulink is shown in Fig. 5.

The simulation is done to mimic a flight mission to evaluate the accuracy and robustness of the controllers under extreme wind disturbance. The overview of the flight mission is to pick a pipe from a target position and place in the desired position. The simulations of the flight mission is structured into smaller objectives, mirroring the approach that will be taken in the actual experiment. This segmentation enables a more realistic and manageable simulation process, aligning with the methodology of the real-world experiment.

The quadcopter is initially set to hover at its initial position and then move towards the $X-Y$ coordinates of the pipe while maintaining the hover altitude. The quadcopter is then made to descent to a desired height and grasp the pipe load, at this stage the weight of the pipe is added to the slung load. The wind disturbance is then applied as the quadcopter grabs the pipe for take-off. The quadcopter is then made to ascent back to the hover altitude along with the slung load and move to $X-Y$ coordinates of the desired pipe drop position. The quadcopter is then made to drop the load after descending to the desired drop location. It is then set to takes off and go backs to the initial position and then land under wind disturbance.

**Fig. 6:**
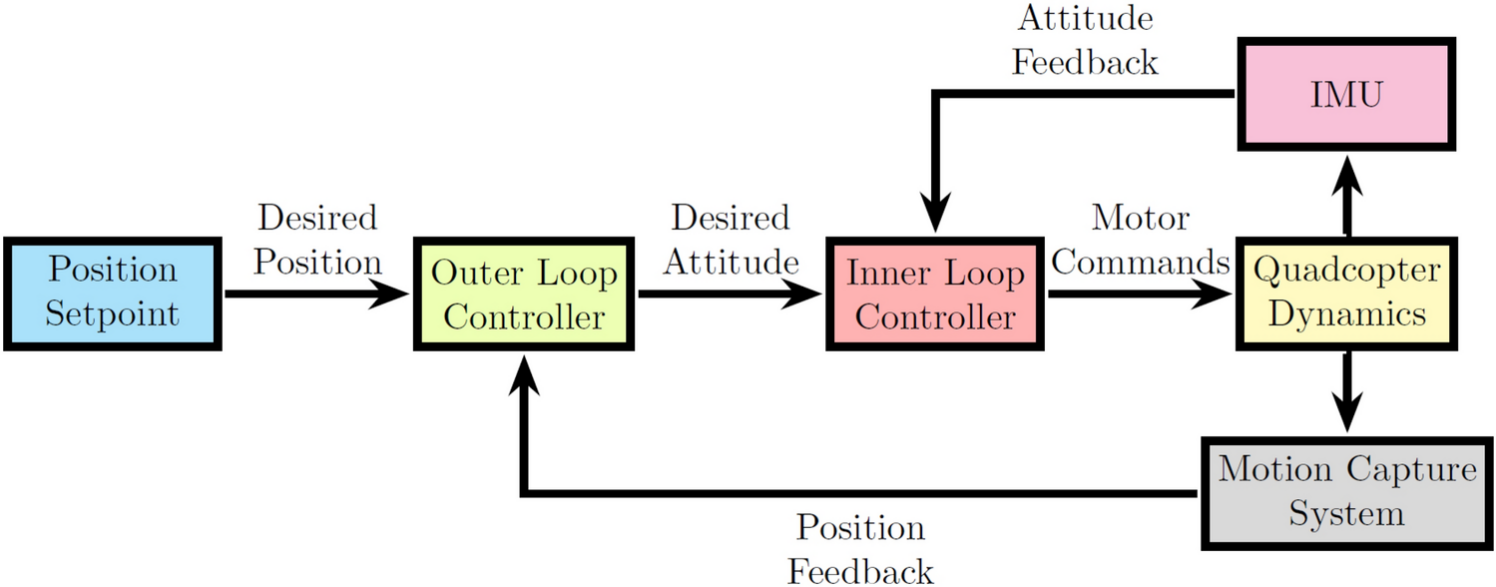
Quadcopter control architecture for both simulations and experiments.

The simulations are conducted using three controllers, namely DNN-MRFT based PID controller^20,21^, the SO-BFBEL controller^18^ and the FSMC controller with the structure is shown in Fig. 6 and they are implemented to control all 6DOF. The flight control for the UAV needs to consider the parametric uncertainties that are caused by the hardware and the non-linear dynamics of the system. The conventional PID controllers are generally used by manually tuning the controller for each parameter and fine tuning till the desired performance is achieved. This process is generally time consuming and inefficient as the PID controllers tuned separately for each parameter may need more fine tuning when all 6DOF are controlled together. To overcome this, the DNN-MRFT based PID controller is chosen and this approach needs the DNN to be trained using the simulated flight data and then the MRFT technique to auto-tune the PID parameters to get the optimal performance^32^. Though the learning of the DNN depends on offline flight data (or simulation data) and further MRFT fine tuning, this method makes the tuning of the PID controller more systemic and consistent compared to the conventional methods.

The FSMC controller used trapezoidal membership function where the $G_e$ and $G_{de}$ control gains are manually tuned. The stability and convergence of the FSMC relied on the $\gamma$ value which is quite sensitive to the system response. Meanwhile, the SO-BFBEL controller did not need any offline training or tuning to implement the controller. The controller is directly initialised with zero initial weights and its initial number of fuzzy layers are set to 1. The self organising (SO) algorithm automatically increases and decreases the required number of fuzzy layers with suitable fuzzy range in real time which simplifies the design process significantly. This setup also makes the implementation of this controller much easier and with less effort. The maximum number of layers are limited to 5 and the minimum is set to be 3 once it crosses that number. This controller has been previously simulated and validated on a quadcopter platform to perform trajectory tracking and showed superior results. But for this paper, the quadcopter includes the custom two finger gripper and a external unstable load that can swing and disturb the CoG of the UAV which challenges the overall stability of the system to perform the aerial manipulation task. This simulation intends to test its capabilities and performance to analyse the utility of the SO-BFBEL controller further with its suitability to aerial manipulation for unmodelled disturbances and uncertainties especially in windy environment.

**Fig. 7:**
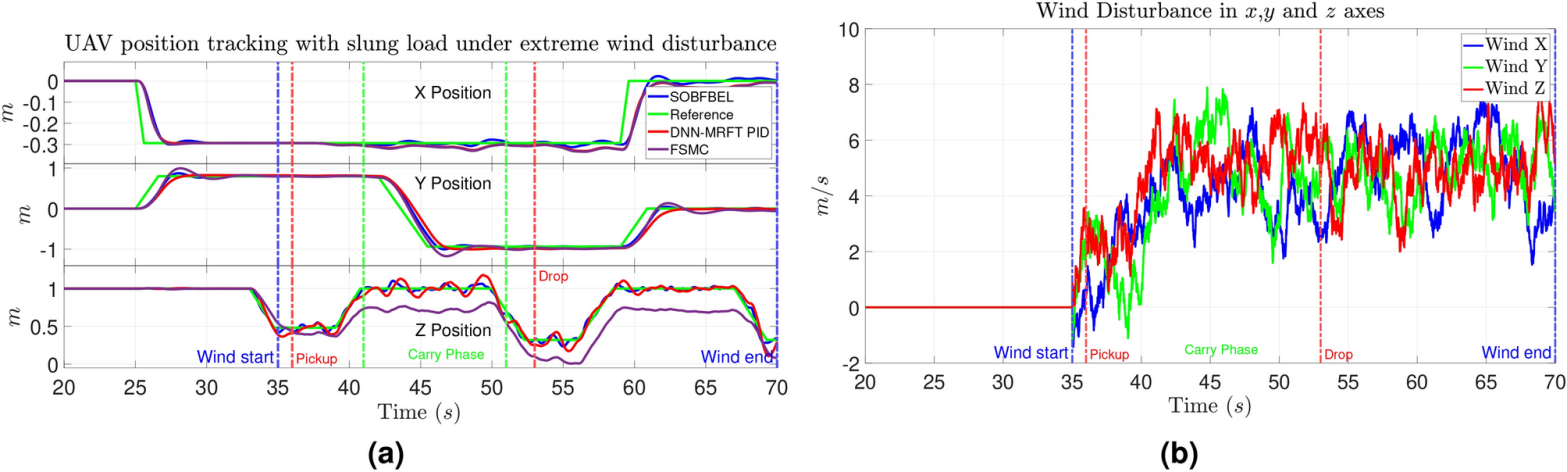
(**a**) Simulation results of quadcopter position tracking under wind disturbance; (**b**) Simulated wind disturbance applied to the system.

The simulation results of the three controllers under the wind disturbance are given in Fig. 7a with the respective the wind disturbance as shown in Fig. 7b. The average root mean square error (RMSE) values of the three controllers are given in Table 1 for comparison.

**Table 1:** Root Mean Square Error (RMSE) comparison between DNN-MRFT based PID, SO-BFBEL and the FSMC controller.

Parameter	DNN-MRFT PID	SO-BFBEL	FSMC
RMSE of position (cm)			
X Position	4.55	4.34	4.69
Y Position	16.13	12.36	13.1
Z Position	6.15	3.10	21.12

The results shows that the DNN-MRFT based PID and the SO-BFBEL controller could successfully control the UAV under wind disturbance with varying payload but the FSMC was unable to perform satisfactorily. The DNN-MRFT based PID performed much better than the FSMC to handle the wind disturbance and changing payload but So-BFBEL controller had much better performance. The main reason behind the performance of the PID controller is the DNN-MRFT tuning mechanism which systematically tuned the gains for the environment it is intended to be operated. So the offline training method proved effective as it fine tuned the controller to be able to get the optimal performance.

For this simulation, the FSMC is tuned to work under wind disturbance with the condition that it should still work when there is no wind so it is gain scheduled to switch between the conditions. The results shows that the FSMC performed poorly despite being adaptive and it is mainly due to its sliding surface mechanism which smoothens the control response but dampens the performance under wind disturbance. The FSMC performed satisfactorily under ideal conditions and with changing payload but failed under the the wind disturbance. The $G_e, G_{de}$ and $\gamma$ control gains of the FSMC needs to be set-up with gain scheduling for ideal conditions and under wind disturbance. The main drawback is that the difference in the control gain values are of very wide margin, that is, the gains used for position tracking without wind disturbance failed when the wind disturbance is applied so finding the exact gains for every disturbance and uncertainty is not feasible all the time.

The results shows that the SO-BFBEL controller shows better performance for the *X* position control and significantly better performance for *Y* and *Z* position control. It can also be noted that the effect of load swing and load weight is much less using the SO-BFBEL controller than the other two controllers while performing the flight trajectory. These results prove that the SO-BFBEL can handle the quadcopter up to 5$\hbox {m\,s}^{-1}$ of wind disturbance with turbulence of up to 12$\hbox {m\,s}^{-1}$ which are quite extreme and shows the controller is capable of performing the mission in real experiments in extreme wind. To quantify the metrics, the SO-BFBEL controller performed over [5%, 24%, 50%] better than the DNN-MRFT based PID controller for [*X*,  *Y*,  *Z*] position tracking. Since the FSMC controller did not achieve satisfactory altitude control, which is critical for the mission, despite the gain scheduling, it is deemed unsuitable for further experimentation.

## Experimentation

Experiments are conducted to perform pickup and placement of a metal pipe using a quadcopter attached with a gripper. Multiple experiments are conducted for different scenarios to test the practicality of aerial grasping for pipe placement and to evaluate the performance of the controllers. The quadcopter is set to follow a predefined way-points trajectory to achieve the desired goal. In this section, the experimental setup is introduced, followed by the experimentation process and the results along with their analysis.

For this research, a Quanser Qdrone quadcopter UAV^23^ is used as the base platform. A sliding rail gripper mechanism using single actuator is attached to the base of the quadcopter in alignment with the CoG of the quadcopter. Four elevated landing gears are attached to prevent the gripper from hitting the floor and for the safe landing of the quadcopter. The total weight of the quadcopter including the gripper and the landing gear is 1370 g. A metal pipe of 66.4 cm length and 2 cm diameter is used as the pipe load. The pipe is attached with four motion capture markers to track its position and attitude. Two small bases are attached to the pipe ends to prevent it from rolling and moving during the experimentation. The total weight of the pipe is 121.8 g.

The OptiTrack Motion Capture System (MCS) is used to obtain the position of the quadcopter, as well as the position and the angle of the pipe. The roll and pitch attitude of the quadcopter is obtained from an inertial measurement unit (IMU) sensor mounted on the quadcopter. The controller is run on the onboard processor and the position data is transmitted through WiFi from the MCS. The experiment is done using Matlab Simulink software with an interface provided by Quanser. The experimentation is done in a closed space with the total dimension of $8.54 \times 8.46 \times 3.46$ m $(L\times W\times H)$ where the available flight area is $2.7 \times 3.5 \times 1.5$ m

A 42” (1050 mm) IGMA Vega heavy duty industrial drum fan^33^ is used to generate wind disturbance up to 6.8$\hbox {m\,s}^{-1}$ and their wind profile is given in^18^. The closest and farthest distances between the quadcopter and the industrial fan are 1.5 m and 2.5 m, respectively. The industrial fan is tilted at 88 degrees and it is positioned to create wind disturbance along the *Y* axis. The industrial fan creates strong wind inside the closed indoor flight area and causes the direction of wind to flow chaotically which recreates a scenario of windy environment and an environment similar to an incomplete construction site of a high rise building.

### Aerial manipulation test methodology

Both the SO-BFBEL and the DNN-MRFT based PID controllers are implemented to control all 6DOF and they are utilised to perform pipe pickup from an elevated platform and to place it at the desired location. The elevated platform is used to mimic a situation where the access is restricted by ground. The quadcopter altitude set points for pickup and placements are pre-calculated based on the landing gear and gripper dimensions. The quadcopter is then programmed to execute the flight mission in the following stages:

1. Set the quadcopter to take-off and hover at [0,0,1] as the initial X–Y–Z position up to 25 s with the gripper in open position.
2. Go to the pipe pickup X–Y coordinates.
3. Descent down to the pickup altitude and make sure the pipe position is between the gripper fingers.
4. Grasp the pipe by closing the gripper.
5. Ascent back to the initial hover altitude while maintaining the pickup X–Y coordinates.
6. Take the pipe to the designated X–Y coordinates for drop.
7. Descent down to the drop altitude.
8. Drop the pipe by opening the gripper.
9. Ascent back to the initial hover altitude while maintaining the drop X–Y coordinates.
10. Go the initial X–Y–Z position and land the quadcopter.

**Fig. 8:**
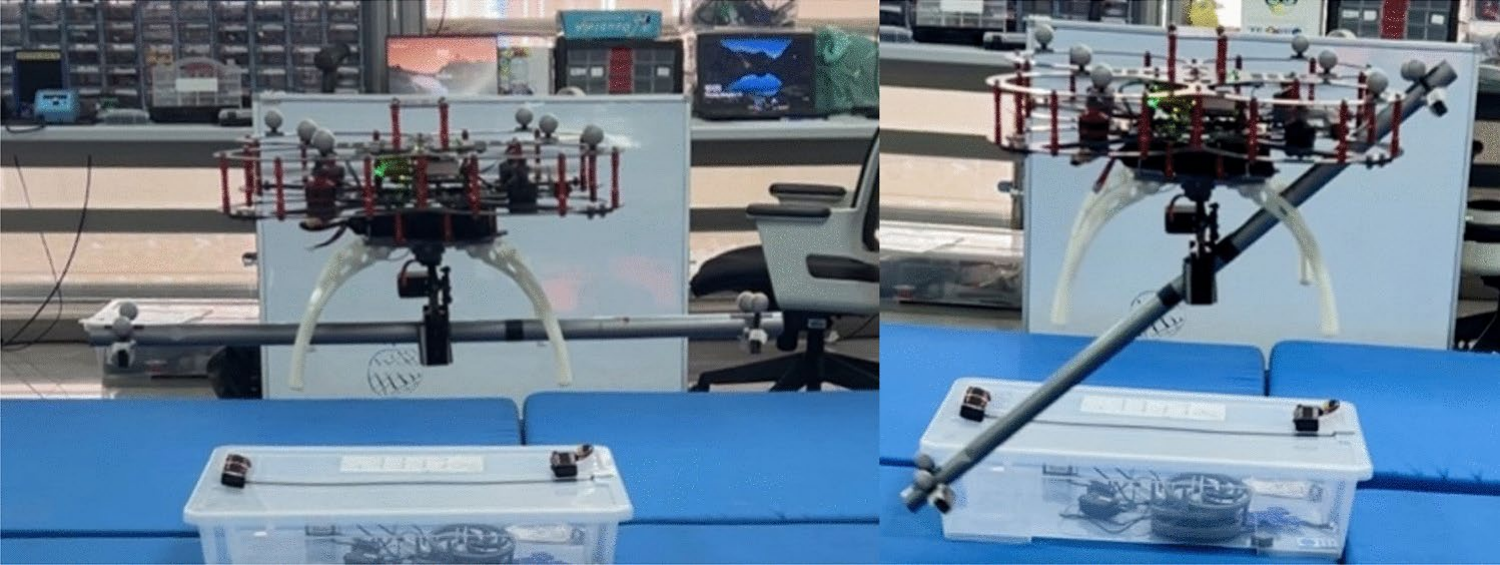
Comparison of aerial manipulation for balanced (left) and unbalanced (right) pipe position.

The experiments are conducted for three scenarios to demonstrate the capability of the aerial manipulation system and to evaluate the accuracy and robustness of the controllers. First, the pipe is grasped at its CoG for balanced load. Second, the pipe is grasped slightly away from the CoG for unbalanced load. Figure 8 shows the comparison of flight with balanced (left) and unbalanced (right) pipe load. Finally, the pipe is grasped at its CoG and it is then carried and delivered under wind disturbance as shown in Fig. 2b. All the flight tests are done using two controllers, namely DNN-MRFT based PID controller, and the SO-BFBEL controller. The three scenarios are described below.

#### Case 1: Balanced pipe load

In this scenario, the exact CoG position of the pipe is set as the reference setpoint for the X-Y position tracking for the pickup. This scenario is the ideal case where the exact CoG of the pipe is known and to evaluate the accuracy of the controllers to perform accurate pickup of the pipe. In this setup, the accuracy of the controllers can be evaluated by the horizontal angle of the pipe during the mission as smaller angle will translate to more balanced pipe.

The quadcopter is then set to execute the flight mission with both the controllers. This experiment is repeated six times consecutively for the SO-BFBEL controller and more than fifteen times for the DNN-MRFT based PID controller. We had to repeat the experiment many times using the DNN-MRFT based PID controller to get six satisfactory results as the controller missed the CoG mark more often. This is done to have a fair performance comparison between the SO-BFBEL and the DNN-MRFT based PID controller.

**Fig. 9:**
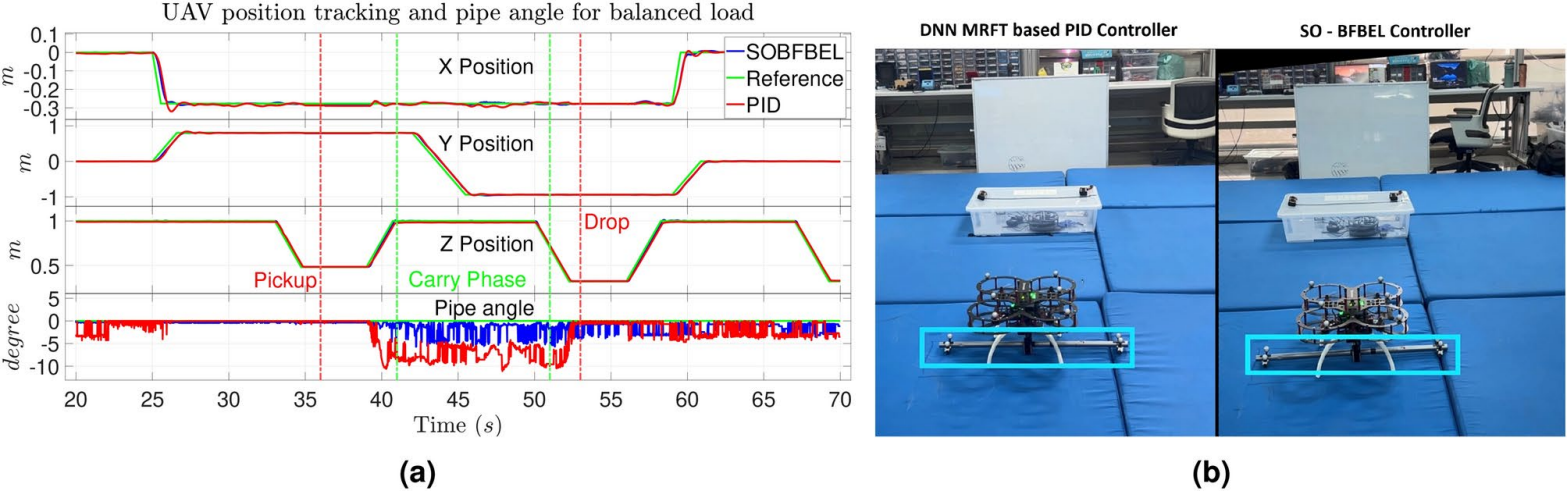
(**a**) Quadcopter position tracking and pipe angle; (**b**) Pipe placement with balanced load using the DNN-MRFT based PID controller (on left) and the SO-BFBEL controller (on right) for Case 1.

The results of the quadcopter position tracking and horizontal angle of the pipe using both the controllers for balanced load is shown in Fig. 9a. Both the controllers achieves this mission successfully as shown in Fig. 9b. The average RMSE values of six flight tests for both the controllers are given in Table 2.

**Table 2:** RMSE comparison for all the cases. Here, PID denotes the DNN-MRFT based PID controller and SO denotes the SO-BFBEL controller.

Cases	Balanced (cm)		Unbalanced (cm)		Wind (cm)	
	PID	SO	PID	SO	PID	SO
X pos	2.12	1.89	2.23	1.76	3.64	2.17
Y pos	5.17	5.04	5.06	4.73	9.91	5.54
Z pos	2.19	1.12	2.24	1.11	2.27	1.25
X pick up	1.03	0.48	0.56	0.62	0.79	0.64
X carry	0.73	0.44	1.12	0.47	1.67	1.48
Y carry	8.15	7.68	8.25	7.38	11.25	10.00
Z carry	1.74	0.67	1.83	0.67	1.93	0.90
X drop	0.94	0.37	1.00	0.51	2.54	1.59
Y drop	0.30	0.31	0.45	0.62	4.90	2.54
Average pipe angle (horizontal) (degree)						
Carry	7.24	1.94	36.40	37.71	32.36	11.49
Average computation time during flight (s)						
Time $(e^{-4})$	8.7	8.5	8.5	8.3	8.6	8.5

In Table 2, the first three parameters namely, “X pos, Y pos and Z pos” denotes the performance for the full flight of the mission followed by the different segment of the activities during the mission as marked in the Fig. 9a. The notation “X pick” denotes the performance during the “Pickup” activity and the next three parameters, “X carry, Y carry and Z carry” denotes the performance during the “Carry Phase” activity . The next two parameters “X drop and Y drop” denotes the performance during the “drop” activity. And finally the average pipe angle and average computation time during flight denotes the average horizontal angle of the pipe during the “Carry Phase” and the average computation time per iteration of the control system for both the controllers.

For the balanced pipe load, the horizontal angle of the pipe during the “Carry Phase” is much smaller for the SO-BFBEL controller at 1.94 degree compared to the DNN-MRFT based PID controller at 7.24 degree which is more than 3.7 times higher than the SO-BFBEL controller. This is because the average X position tracking error during “Pickup” for SO-BFBEL controller is at 0.48 cm whereas for the DNN-MRFT based PID controller, it is at 1.03 cm, which is more than two times the error margin of the SO-BFBEL controller. The consequence of this error margin during pick reflects on the placement position of the pipe. The X position tracking error of the pipe during its placement for SO-BFBEL controller is at 0.37 cm whereas for the DNN-MRFT based PID controller, it is at 0.94 cm which is more than 2.5 times the error margin of the SO-BFBEL controller.

The SO-BFBEL controller achieves lower RMSE values in *XYZ* position tracking throughout the full flight compared to the DNN-MRFT based PID controller which highlights its accuracy in position tracking. The SO-BFBEL controller rapidly adapt to handle the disturbance caused by the swing of the gripper when it is moved in *XY* direction whereas the DNN-MRFT based PID controller overshoots for the same action and it is quite noticeable for *X* position at 27 s and 60 s in Fig. 9. The SO-BFBEL controller has much better trajectory tracking performance in the “Carry Phase” as it can adapt to handle the uncertainties caused by the pipe load during the mission.

Overall, the SO-BFBEL controller outperforms the DNN-MRFT based PID controller across all parameters, as indicated by lower RMSE values. This demonstrates the feasibility and practical use of the quadcopter for pickup and placement of pipes using the SO-BFBEL controller. Repeated experimentation consistently demonstrated the greater accuracy in trajectory tracking using the SO-BFBEL controller which helps to accomplish the mission more efficiently compared to the DNN-MRFT based PID controller.

#### Case 2: Unbalanced pipe load

In this scenario, the reference X–Y position setpoint for the pipe pickup is set up to 2.5 cm away from the CoG of the pipe. This scenario is designed to mimic a situation where the exact CoG of the pipe is not available and its accuracy is tolerable by few cm error margin. This scenario is to evaluate the robustness of the controllers to perform reliable mission where the unbalanced pipe load disturbs the CoG of the quadcopter which directly affects the performance and stability of the system and ultimately increase the risk of mission failure. In this setup, the robustness of the controllers can be evaluated by the performance of the quadcopter during the mission as smaller error will translate to more stable and robust performance.

**Fig. 10:**
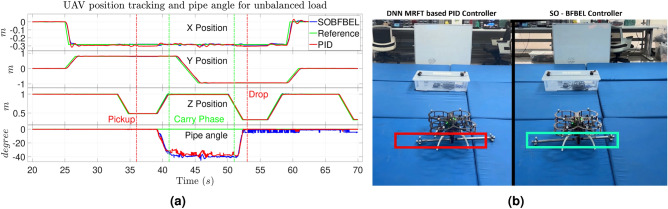
(**a**) Quadcopter position tracking and pipe angle; (**b**) Pipe placement with unbalanced load using the DNN-MRFT based PID controller (on left) and the SO-BFBEL controller (on right) for Case 2.

Similar to the first scenario, the quadcopter is set to execute the flight mission with both the controllers. This experiment is repeated five times consecutively with the SO-BFBEL controller and several times with the DNN-MRFT based PID controller. The top five results of DNN-MRFT based PID controller are chosen where the horizontal angle of the pipe lies between 3538 degree, to avoid unfair comparison. The results of the quadcopter position tracking and horizontal angle of the pipe using both the controllers for unbalanced load is shown in Fig. 10a. The SO-BFBEL controller completes this mission successfully whereas the DNN-MRFT based PID controller fails to place the pipe in the target location as shown in Fig. 10b.

The average RMSE values for unbalanced load is given in Table 2. Here, the SO-BFBEL controller maintains a consistent average RMSE for the quadcopter position during the “Carry Phase” despite the unbalanced load. Whereas the performance of the DNN-MRFT based PID controller deteriorated, especially along the *X* axis where the unbalanced load directly applies the disturbance. The average horizontal angle of the pipe during the “Carry Phase” for the SO-BFBEL and the DNN-MRFT based PID controller are measured at 37.71 degree and 36.4 degree respectively. This implies that the similar unbalanced load setting is applied to both the controllers for comparison. The SO-BFBEL controller achieves superior overall position tracking, though the DNN-MRFT-based PID controller shows a slightly better average in the “X pick.” This difference arises because the average value for DNN-MRFT based PID controller is calculated from its top five best tracking results, selected from a broader dataset that includes both high and low performance. Whereas for the SO-BFBEL controller the average was taken from five consecutive flights which all had more consistent results. This highlights that the SO-BFBEL controller can performs better to handle uncertainties in realtime during the mission and it is more reliable and robust than the DNN-MRFT based PID controller.

#### Case 3: Transport under extreme wind disturbance

In this scenario, the exact CoG position of the pipe is set as the reference pickup setpoint for the X–Y position and the industrial fan is turned on once the descent to grasp the pipe at its CoG is successful. This mimics the situation where the quadcopter picks the pipe successfully but then there is a severe gust or turbulent wind in open field or due to a open window or door or a missing wall in a high rise building under construction. This scenario is to evaluate the accuracy and robustness of the controllers to perform the mission. In this setup, the performance of the controllers can be evaluated by the horizontal angle of the pipe during the mission as well as the position tracking error values. Smaller error will translate to more stable flight and lower pipe angle will indicate more balanced pipe. This experiment is repeated two times with the SO-BFBEL controller and several times with the DNN-MRFT based PID controller until we got two successful pickup and placement of the pipe load.

**Fig. 11:**
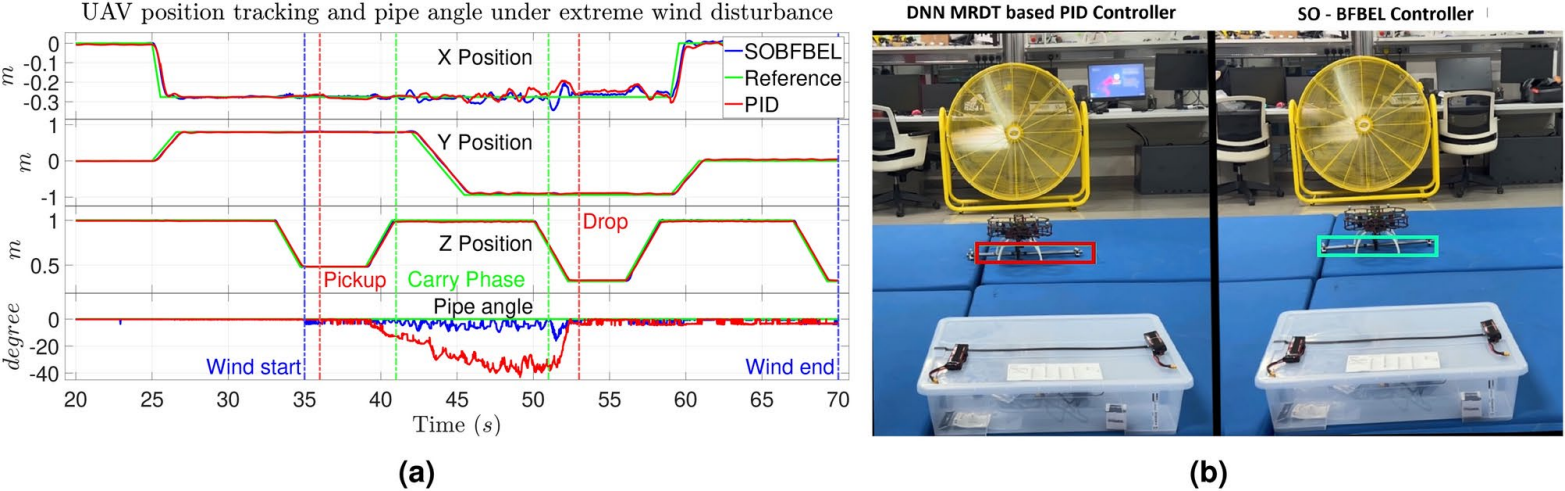
(**a**) Quadcopter position tracking and pipe angle; (**b**) Pipe placement under wind disturbance using the DNN-MRFT based PID controller (on left) and the SO-BFBEL controller (on right) for Case 3.

The industrial fan was turned on at maximum speed at 35s as marked as “Wind start” in the Fig. 11a and it is turned off at the end of the mission. The industrial fan is placed at 150 cm (1.5 m) in-front of the pipe drop location along the *Y* axis. Similar to the previous two scenarios, the quadcopter is set to execute the flight mission with both the controllers. The results of quadcopter position tracking and the horizontal angle of the pipe using both the controllers are shown in Fig. 11a. Similar to the previous case, the SO-BFBEL controller completes this mission successfully whereas the DNN-MRFT based PID controller fails to place the pipe in the target location as shown in Fig. 11b.

Unlike the previous scenarios, the wind disturbance along the *Y* axis significantly affects the quadcopter due to the larger surface area of the pipe and its impact on the position of the quadcopter increases as it moves towards the drop location. The wind disturbance applied to the quadcopter is maximum at the drop coordinates and minimum at the pickup coordinates. This flight test was more challenging as the DNN-MRFT based PID controller failed to contain the quadcopter oscillation most of the time as it got closer to the fan and this resulted in the pipe dropping pre-maturely whereas this problem was not faced using the SO-BFBEL controller.

From the results, it can be seen that the horizontal angle of the pipe during the “Carry Phase” is smaller for the SO-BFBEL controller than the DNN-MRFT based PID controller. This is because the SO-BFBEL controller was more stable in its position tracking which reduced the of shaking of the quadcopter which ultimately helped in maintaining the pipe position for a longer time. Whereas the DNN-MRFT based PID controller was not as stable in maintaining the quadcopter position especially under wind disturbance which caused the pipe to move more and slip beyond the grasping area of the gripper which ultimately led to become an unbalanced load during the mission. This behaviour significantly increased the rate of mission failure using the DNN-MRFT based PID controller.

The position tracking error of the quadcopter for the whole mission using the SO-BFBEL controller is much lower than the DNN-MRFT based PID controller for all three position control. To quantify the metrics, the SO-BFBEL controller performs over [11%, 21%, 40%], [2.5%, 6.5%, 44%] and [49%, 50%, 45%] better for the [*X*,  *Y*,  *Z*] position control than the DNN-MRFT based PID controller for “balanced”, “unbalanced” and “wind” scenarios respectively. From these results it can be noted that the SO-BFBEL performs significantly better when disturbances and uncertainties are involved. These numbers are consistent with the simulation results where the difference in performance is up to $50\%$.

### Analysis of the controller characteristics

In this section we analyse the characteristics of both the controller for all the three cases with respect to the battery voltage and discharge rates. While testing for the three cases of flight test, we had to repeat some flight test several times using the DNN-MRFT based PID compared to the SO-BFBEL controller. To diagnose the main cause of this problem, all the flight data using the DNN-MRFT based PID controller were analysed. Through numerous flight data, we observed that not all batteries discharge at same rate even if it is the same model with same specification. The discharge rate depends on the usage frequency of each battery and they are difficult to identify as they are generally used in random order. Even if the batteries are numbered and used in a specific pattern it is still difficult to tune the controllers for each battery profile every time as this can affect the performance of the controller and makes the tuning more cumbersome. The battery profile of the individual flight for all the three cases are plotted to analyse the effects of the battery discharge rate on the flight mission.

**Fig. 12:**
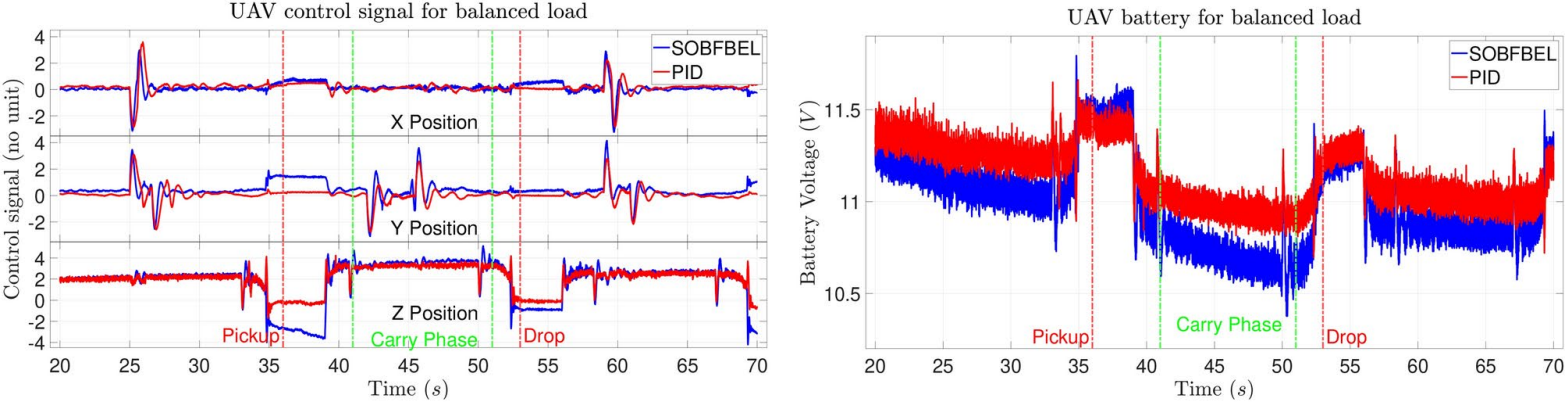
Control signal comparison (left) and battery discharge rate (right) for balanced pipe load (Case 1).

The control signal comparison and the battery discharge rate for balanced pipe load for Case 1 is shown in Fig. 12. This plot belongs to the trajectory tracking results shown in Fig. 9. It is observed that the initial battery voltage for the DNN-MRFT based PID controlled flight is marginally higher than the SO-BFBEL controlled flight test. Here, the battery discharge rates are different where the battery discharge rate of the SO-BFBEL controlled flight is higher than the DNN-MRFT based PID controlled flight test. This behaviour is quite noticeable during the “Carry Phase”. Another strange behaviour is that the battery discharge rate of the SO-BFBEL controlled flight is higher till the end of the “Carry Phase” and becomes similar to the DNN-MRFT based PID controlled flight after that. This clearly shows the uncertain discharge rates of each individual battery which are beyond our reasonable control.

A trim value is bias which is manually adjusted to be slightly below the minimum threshold value at which the UAV starts to take-off from the ground and it is generally added to the thrust control signal of the controller to avoid sudden spike in control signal during take-off which may cause overshoots. This trim value is added to both the SO-BFBEL and DNN-MRFT based PID controller for all the experiments. From Fig. 12, it can be noted that the control signal from the SO-BFBEL controller for the altitude (Z position) decreases more during the “Pickup” and “Drop” activities as it is adapting towards smaller error margin by reducing the control signal gradually which in-turn reduces the battery consumption compared to the DNN-MRFT based PID controller. This is due to the SO-BFBEL controller adapting to go below the trim value in real-time and it did not have any overshoot when the UAV is commanded to take-off again. This is not the case with the DNN-MRFT based PID control as it overshoots higher than the intended altitude leading to unnecessary oscillation which can compromise the mission so its integral gain (I) output is limited. The ability of the SO-BFBEL controller to go below the trim value while landing and recovering back to hover without any penalty led to the increased the battery voltage for a short interval despite the lower starting voltages. It also resulted in the SO-BFBEL controlled flights to have a higher battery voltage at the end of the mission compared to the DNN-MRFT based PID controlled flight despite starting with a lower voltage for the same mission.

**Fig. 13:**
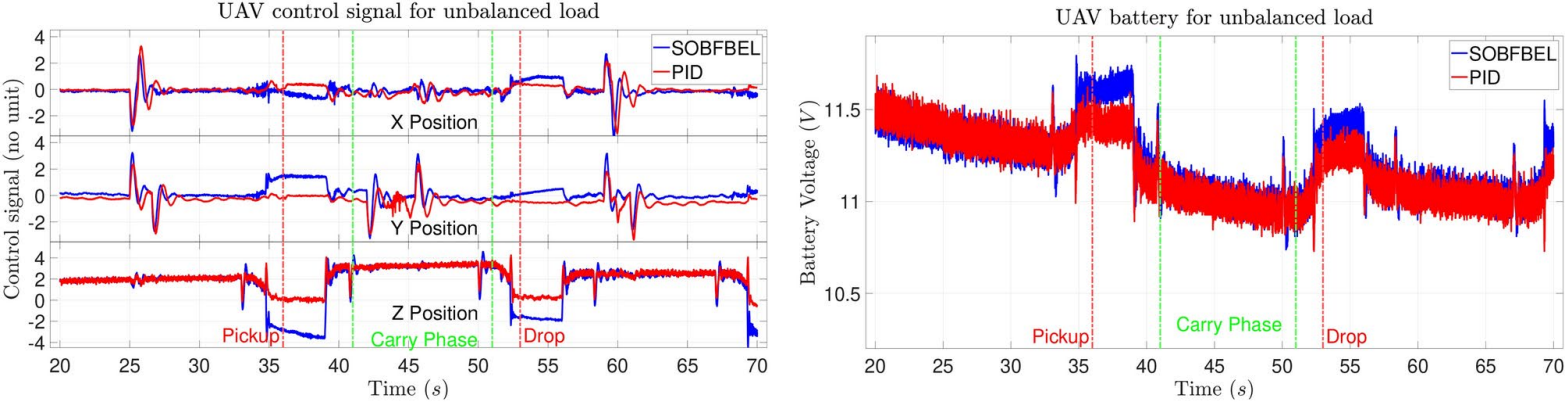
Control signal comparison (left) and battery discharge rate (right) for unbalanced pipe load (Case 2).

The control signal comparison and the battery discharge rate for unbalanced pipe load for Case 2 is shown in Fig. 13. This plot belongs to the trajectory tracking results shown in Fig. 10. For this case, the initial battery voltage using both the controllers are at the same level. It can be seen that the battery discharge rate of both the batteries are almost the same. Similar to the previous case, the control signal from the SO-BFBEL controller for the altitude (Z position) decreases more during the “Pickup” and “Drop” activities which in-turn reduced the battery consumption noticeably compared to the DNN-MRFT based PID controller. This behaviour resulted in higher battery voltage at the end of the mission for the SO-BFBEL controlled flight compared to the DNN-MRFT based PID controlled flight for the same battery level.

**Fig. 14:**
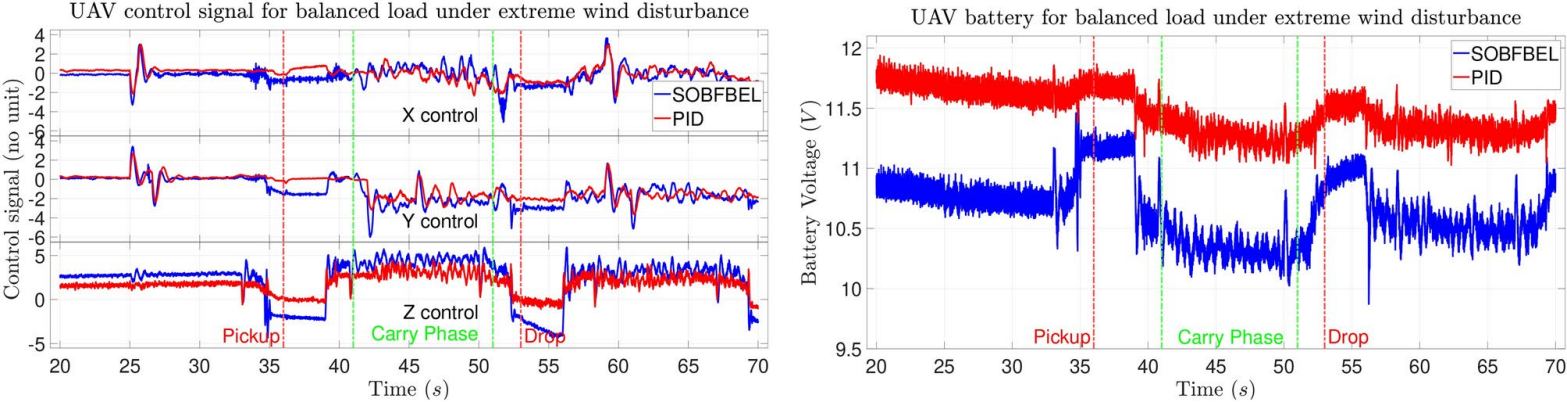
Control signal comparison (left) and battery discharge rate (right) under wind disturbance (Case 3).

The control signal comparison and the battery discharge rate for balanced pipe load under extreme wind disturbance for Case 3 is shown in Fig. 14. This plot belongs to the trajectory tracking results shown in Fig. 11. For this case, the initial battery voltage using both the controllers are at very different levels. The battery level of the DNN-MRFT based PID controlled flight was at its full charge whereas the battery level of the SO-BFBEL controlled flight was at very low charge. It can be seen that the battery discharge rate of both the batteries have very different characteristics. The discharge rate of the battery with full charge was more gradual throughout the flight whereas the discharge rate of the battery with low charge started to show more oscillatory or unstable discharge rate, especially from the “Carry Phase” onwards. It was observed that, at lower battery levels, the performance of the DNN-MRFT based PID controlled flight was noticeably worse and it was not a coincidence that all the successful flight using the DNN-MRFT based PID controller had full or almost full charge of the battery. This was not the case using the SO-BFBEL controller as the battery level or its discharge rate did not have any impact on its performance. The SO-BFBEL controller compensated the effect of oscillatory battery discharge rate by producing appropriate control signals in real time which completely negated its effects whereas the DNN-MRFT based PID controlled flight was observed to oscillate to a point it becomes uncontrollable and unstable.

The SO-BFBEL controller showed remarkable adaptability and resilience to uncertainties and delivered more consistent performance throughout the test. Whereas the DNN-MRFT based PID controller failed on more occasions and suffered performance degradations due to battery and ground effect which resulted in repeating the test several times till satisfactory results were achieved. This resulted in consuming significant amount of time re-tuning and fine-tuning the controller gains at the beginning and it was later minimised by using fully charged batteries.

To summarise all the results, the proposed aerial manipulation system is assessed for precision, adaptability to environmental variations, reliability and overall operational efficiency for pipe pickup and placement. The proposed aerial manipulation system proved to be more robust and practical for the task of aerial manipulation in the context of civil engineering works. The performance of the proposed system is significantly better, reliable and efficient using the SO-BFBEL controller than the DNN-MRFT based PID controller. The numerous flight tests and their average RMSE metrics clearly demonstrates the superior performance of the SO-BFBEL controller. In addition, the computation cost and battery saving characteristics of the SO-BFBEL controller makes it more efficient than the DNN-MRFT based PID controller for all the scenarios. The complete summary of the characteristics analysis of both the controllers are highlighted in Table 3.

**Table 3:** Comparison of DNN-MRFT based PID and SO-BFBEL controller characteristics for aerial manipulation.

Criteria	DNN-MRFT based PID controller	SO BFBEL controller
Training requirements	Requires pre-training of DNN using flight data for effective PID tuning	No pre-training is required
Parameter Tuning	MRFT tuning with additional manual adjustments; complex due to DNN integration	Self-optimising, uses adaptive evolutionary approach without manual intervention
Tuning Complexity	Difficult; MRFT algorithm must be rerun for experimental flight tuning/re-tuning.	Easy; self organising algorithm automatically adjusts controller parameters in real-time
Real-time Adaptation	None; DNN is trained under ideal conditions and requires MRFT re-tuning for environmental changes	High; dynamically adapts in real-time with self-organising adjustments for optimal performance
Deployment Ease	Difficult; requires pre-training and MRFT testing to get experimental gain values for each parameter which requires more effort and time to implement	Easy; adapts rapidly for all the parameters in real time which requires less effort and time to implement
Tolerance to Uncertainties	Low; significantly affected by uncertain battery discharge rates and less tolerant to wind disturbance and ground effect	High; unaffected by uncertain battery discharge rates and more tolerant to wind disturbance and ground effect
Computation cost	Low; The real-time computation cost is low and it is the main reason why it is widely used.	Low; The real-time computation cost is low with much superior performance so it can be considered as a suitable replacement for the PID controller.
Overall Implementation Difficulty	High; complex DNN pre-training and occasional manual or MRFT re-tuning increases the implementation effort	Easy; no pre-training and self organising nature significantly reduces the implementation effort

## Conclusion

In conclusion, this paper introduced a novel aerial manipulation system designed for the precise handling of long objects, particularly in constrained spaces and windy environment. The effectiveness of the proposed system is demonstrated through comprehensive experimentation, highlighting its applicability for pipe handling tasks, particularly in civil engineering contexts. The SO-BFBEL controller exhibited a reliable and superior performance compared to the DNN-MRFT based PID controller, particularly in mitigating wind effects and uncertain battery discharge rates across diverse scenarios. The SO-BFBEL controller was much easier to implement and performed consistently whereas implementing the PID was laborious and performed inconsistently under disturbances. Additionally, the lower computational cost of the SO-BFBEL controller, along with its adaptive characteristics to go below the trim value and recover instantly, helped conserve battery life during the pick-up and drop-off phases which can potentially increase the flight time. Overall, this work advances aerial manipulation technology and presents a promising solution for practical applications across industries and it can be further explored using soft grippers.

## Discussion

The proposed aerial manipulation system offers a practical and scalable solution, particularly suited for swarm operations. A reliable controller is essential to ensure accurate object handling, as failure to grasp the object at the designated point could compromise the mission. The SO-BFBEL controller presents a strong alternative to the conventional PID controller, offering straightforward setup and high adaptability to uncertainties that may not be easily modelled. Additionally, it achieves this without incurring any computational penalty, making it even more compelling for real-time applications. Future work could explore further by expanding this system to a swarm of mid- to large-sized quadcopter UAVs, where each UAV can generate wake and wind disturbances that may impact each other. Addressing these disturbances will be critical for maintaining overall stability and ensuring the operational integrity of the swarm. In addition, it is observed that using custom gripper is more suitable and cost effective as the gripper can be swapped to suit for the intended application whereas, sophisticated grippers that are more versatile come with additional cost to computation and weight that can lower the flight time which can directly increase the operational cost. For our future scope, we would like work towards integrating a closed loop flexible manipulator for aerial manipulation that can overcome these challenges and explore the concepts of cooperative manipulation^34^ and multi-robot grasp coordination^35^ with respect to aerial manipulation in swarm environment. It can also be explored further for predicting remaining useful life for long term commercial operation^36^.

## Data Availability

All data generated or analysed during this study are included in this published article.
